# Acrylamide and bisphenol A: two plastic additives increase platelet activation, via oxidative stress

**DOI:** 10.3389/fphar.2025.1526374

**Published:** 2025-04-30

**Authors:** C. F. Burgos, D. Méndez, S. Quintana, S. Gonkowski, A. Trostchansky, M. Alarcón

**Affiliations:** ^1^ Department of Physiology, Faculty of Biological Sciences, Universidad de Concepción, Concepción, Chile; ^2^ Thrombosis Research Center and Healthy Aging, Universidad de Talca, Talca, Chile; ^3^ Department of Clinical Physiology, Faculty of Veterinary Medicine, University of Warmia and Mazury in Olsztyn, Olsztyn, Poland; ^4^ Departamento de Bioquímica and Center for Free Radical and Biomedical Research, Facultad de Medicina, Universidad de la República, Montevideo, Uruguay; ^5^ Department of Clinical Biochemistry and Immunohematology, Faculty of Health Sciences, Universidad de Talca, Talca, Chile

**Keywords:** microplastics, acrylamide, bisphenol, platelets, cardiovascular diseases, platelets and cardiovascular diseases

## Abstract

**Background:**

Since the mid-20th century, the widespread use of plastics has led to the buildup of harmful byproducts in the environment—most notably acrylamide (AA) and bisphenol A (BPA). These chemicals are now commonly detected in human tissues, raising concerns about their potential health effects. While their presence as environmental pollutants is well known, their specific impact on platelet function and the associated cardiovascular risks remains poorly understood.

**Methods:**

To explore how AA and BPA affect platelet physiology, we performed in vitro assays to assess platelet activation and aggregation following exposure to these compounds. We also used bioinformatic tools to identify potential protein targets in human platelets and carried out molecular docking simulations to investigate how AA and BPA interact with key enzymes involved in platelet regulation.

**Results:**

Both AA and BPA exposure led to a significant increase in platelet activation and aggregation, suggesting an elevated risk of thrombosis. Proteomic analysis identified around 1,230 potential protein targets, with 191 affected by AA and 429 by BPA. These proteins are primarily involved in oxidative stress, apoptosis, and signaling pathways regulated by protein kinase C (PKC), p38α-MAPK, and superoxide dismutase (SOD). Molecular modeling further revealed that AA and BPA form stable complexes with several of these enzymes, indicating direct interference with platelet function.

**Discussion and Conclusion:**

Our study shows that AA and BPA can enhance platelet reactivity and aggregation, which are key factors in the development of cardiovascular disease (CVD). By identifying specific molecular pathways and targets affected by these pollutants, we provide new insights into their potential role in promoting thrombotic conditions. These findings highlight the urgent need for greater public health awareness and stronger regulatory efforts to reduce human exposure to AA and BPA.

## 1 Introduction

Since the second half of the last century, plastic has been an essential element in producing all kinds of materials. Unfortunately, due to its slow degradation and accumulation in the ecosystem, its management has become a severe environmental problem. For example, many elements, such as plastic bags, are used on average for 20 min and then discarded only to contaminate the environment (Galloway et al., 2019). From 1950 to date, about 9,000 million tons have been produced, and according to some projections, by 2050 we will reach a figure of 20,000 million (Geyer et al., 2017). The COVID-19 pandemic increased the levels of plastic worldwide due to the massive consumption, misuse, and mishandling of personal protective equipment and single-use plastics (Fadare and Okoffo, 2020; Prata et al., 2020). In addition to being present in practically all ecosystems, plastic pollution is also found in homes, and chronic exposure may cause health problems. This is through synthetic polymer particles ranging from 5 mm to 1 μm, called microplastics (MP). According to the World Wildlife Fund for Nature (WWF), without realizing it, we consume the plastic equivalent of a credit card (5 g) every week (De Wit and Bigaud, 2019). Recently, concern has been expressed about the consequences of repeated exposure to MP on human health (van Raamsdonk et al., 2020).

Plastics can contain many chemicals that are added during manufacturing and/or absorbed from the environment (Koelmans et al., 2021). Chemicals added to plastic modify its properties for high performance, including plasticizers, flame retardants, photo stabilizers, antioxidants, and pigments, among others (Hansen et al., 2013). Both acrylamide (AA) and bisphenol A (BPA) are components utilized in the manufacturing of plastic (Fauser et al., 2022). The AA and BPA can reach humans through various pathways such as water, air, food, or dust, with a bioavailability of 50%–90% (Gayrard et al., 2013; Otles, 2007). Therefore, it is essential to assess the risk of chemicals and plastics entering our bodies on human health.

AA and BPA are widely used in the plastics industry, are significant environmental pollutants, and have been detected in humans (Zhang et al., 2010). This is why a monitoring program has been established in the United States National Health and Nutrition Examination Survey (NHANES), one of whose functions is to detect chemical substances associated with the use or manufacture of plastics in different populations, including AA and BPA(Lang et al., 2008; Melzer et al., 2010; Neil et al., 2012; Zhang et al., 2018). Consequently, studying these chemicals has aroused growing interest in the scientific community and great concern among many governments due to their possible effects on humans (Fei et al., 2010).

The harmful effects of AA in humans include neurotoxicity, reproductive toxicity, immunotoxicity, hepatotoxicity, nerve damage, muscle weakness, and impaired muscle coordination (Geyer et al., 2017; Hahladakis et al., 2018; Zimmermann et al., 2019). BPA is considered an endocrine disruptor capable of mimicking hormones and, therefore, altering the proper functioning of our body and negatively affecting our health. In this sense, it has been reported that it can affect various tissues such as the heart, pancreas, pituitary gland, and brain (Gao and Wang, 2014), producing a large number of diseases such as cancer, obesity, diabetes, and affecting the reproductive, neuroendocrine, and immune systems (Gao and Wang, 2014). Several studies have reported that the toxicity mechanisms of AA and BPA involve oxidative stress and mitochondrial dysfunction (Mori et al., 2022; Nayak et al., 2022).

A population study by the National Health and Nutrition Examination Survey (NHANES) that collected data between 2003 and 2006 showed that both AA and BPA were considered risk factors for Cardiovascular Diseases (CVDs) (Lang et al., 2008; Melzer et al., 2010; Neil et al., 2012; Zhang et al., 2018), suggesting that these compounds can participate in the possible mechanisms associated with the triggering of this disease. CVDs stand as the primary cause of mortality globally, accounting for an estimated 32% of all worldwide deaths. As an example, in the US a staggering statistic reveals that one person succumbs to CVD every 36 s underscoring the paramount significance of its investigation (Control, 2018). The pathogenesis of CVD involves various intricate processes, including heightened inflammatory responses and the formation of intravascular thrombi. Emerging evidence from numerous investigations suggests that platelet activation assumes a pivotal role in the initiation and progression of CVD, exerting influence over both inflammatory cascades and thrombus generation (Willoughby et al., 2002). Consequently, our study endeavors to ascertain the potential impact of AA and BPA on platelet activation and, consequently, their implication in the development of CVD.

Building on the background in this field, the increased use and contamination of MP, and the critical role that oxidative stress and platelets play in CVD, our goal was to identify new and significant connections. We aimed to elucidate the molecular mechanisms underlying the effects of AA and BPA on platelet function and activation.

## 2 Materials and methods

### 2.1 Bioinformatic analysis

#### 2.1.1 Candidate targets collection

To determine the possible effects of AA and BPA on platelets, we searched several Chemical-Protein Interaction Networks databases (STITCH (Szklarczyk et al., 2015), Binding DB (Gilson et al., 2015), and ChemDis (Tung and Wang, 2018). To identify the proteins present in platelets, we used PlateletWeb (Boyanova et al., 2012). We obtained the genes associated with AA or BPA using an online Venn diagram tool.

#### 2.1.2 Biological function and pathway enrichment

The Gene Ontology (GO) analysis and Kyoto Encyclopedia of Genes and Genomes (KEGG) pathway analysis enrichment were performed using two online platforms:SRplot (Luo and Brouwer, 2013) and MonaGO (Xin et al., 2022). Ranked through p-value, the top 10 relevant biological processes and KEGG pathways (p < 0.05) were plotted with the same platforms. GO terms included biological process, cellular component, and molecular function.

#### 2.1.3 Protein-protein interactions (PPIs) network construction

The candidate platelet protein targets were introduced into four databases (BioGRID (Oughtred et al., 2021), IID (Kotlyar et al., 2015), APID (Alonso-López et al., 2019), and IntAct (Orchard et al., 2013)) for the retrieval of Proteins-Proteins interactions. We then selected the most over-represented targets that were present in the platelets. To further analyze the results from these databases, Cytoscape 3.7.2 was used to visualize and analyze the PPI network (Shannon et al., 2003).

#### 2.1.4 *In silico* evaluation of interactions between target proteins,AA and BPA

Protein-ligand docking was performed using the structures available in the Protein Databank of Protein Kinase C (PKC) C1A and C1B domains (2ENN, 2ENZ), MAP Kinase p38 (PDB ID: 1LEZ) (Chang et al., 2002), Cu-Zn Superoxide dismutase 1(SOD1; 2C9V) (Strange et al., 2006), and Mn Superoxide dismutase 2 (SOD2; 5VF9) (Azadmanesh et al., 2017). All structures were prepared to add hydrogens, assign bond orders, fill in missing side chains, and generate protonation states at pH 7.0 ± 0.2 using Maestro (Schrödinger,LLC,New York,NY,2018). The structures of AA (CID: 6579) and BPA (CID: 6623) were obtained from the PubChem database. Before docking simulations, both compounds were prepared using LigPrep (Schrödinger,LLC,New York,NY,2018) to generate the minimized structures and possible ionization states at pH 7.0 ± 0.2. Initially, docking proteinligand was performed for each protein target using Autodock Vina (Trott and Olson, 2010). For the cases of p38, PKC and SOD1, MKK3B, Phorbol 12-myristate 13-acetate (PMA), and Ebselen were included as positive controls (Capper et al., 2018; Chang et al., 2002; Tahara et al., 2009), respectively since they are recognized activators of the function of both proteins with known binding sites. Subsequently, site-directed dockings were performed with Glide (Schrödinger,LLC,New York,NY,2018) using a receptor grid the size of their respective ligands (10-15Å), with an extra-precision (XP) software configuration. Analysis of the interfaces between target proteins and AA or BPA included structural and energetic parameters performed by the same software. Also, a theoretical MM-GBSA ΔG_bind_ was calculated using Prime (Schrödinger,LLC,New York,NY,2018). All images were created using PyMol (version 1.5, DeLano Scientific LLC).

#### 2.1.5 Molecular dynamic simulations of AA and BPA in a phospholipid bilayer

The molecular dynamics simulation was conducted using the Desmond molecular dynamics package (Schrödinger, LLC, New York, NY, 2018), with the application of the OPLS2004 force field to accurately represent molecular interactions. Data collection from 1000 frames along the simulation trajectory allowed for comprehensive analysis. Solvation effects were accounted for during the production phase through the utilization of the SPC (Simple Point Charge) model. The simulation configuration incorporated thirty orthorhombic boxes as boundary conditions, with charge neutrality achieved through system neutralization. Additionally, to emulate physiological ionic conditions, 0.15 M NaCl was introduced. Furthermore, the plasma membrane was added in the simulation using Desmond, with a model of DOPC composition automatically positioned.

In preparation for the simulation, an initial relax model was employed to ensure system equilibration. To introduce variability, the random seed parameter was utilized. Throughout the simulation, no restraints or custom charges were applied, enabling unconstrained molecular exploration. The simulation itself encompassed a timeframe of 600 ns under NPT conditions, with the temperature set to 300 K and the pressure maintained at 1.01 bars to closely mimic physiological conditions.

### 2.2 Validation and functional analysis

#### 2.2.1 Preparation of human platelets

The platelets were obtained as previously described by our group (Recabarren-Leiva et al., 2021). Briefly, two different types of platelet samples, Platelet-Rich Plasma (PRP) and Washed Platelet (WP), were used for aggregation studies and cytotoxic and ROS assays, respectively.

Platelet-rich plasma (PRP) and washed platelets (WP) were obtained from 10 mL of venous blood samples drawn into citrate tubes (3.2%; 9:1 v/v) (Becton Dickinson Vacutainer Systems, Franklin Lakes, NJ, United States) or collected in syringes containing ACD (9:1 v/v) supplemented with 12 mM Theophylline and 0.11 μM prostaglandin E_1_, respectively. The blood samples underwent centrifugation at 240 *g* for 10 min (Eppendorf centrifuge 5,804, Hamburg, Germany), resulting in the separation of PRP. Two-thirds of the supernatant was removed to isolate PRP, while the remaining volume underwent further centrifugation at 650 *g* for 10 min to obtain Platelet-Poor Plasma (PPP). Subsequently, PRP was adjusted with PPP to a concentration ranging between 200 and 300 × 10^3^ platelets/µL using a cell counter (Mindray BC-3000 plus, Shenzhen, China Tarrytown). For the obtention of WP, following centrifugation at 240 *g* for 10 min, the supernatant was subjected to a second centrifugation at 650 *g* for 10 min, yielding a pellet of platelets. This pellet was then resuspended in 500 µL of washing buffer (137 mM NaCl, 2.7 mM KCl, 2 mM MgCl_2_, 9.5 mM NaHCO_3_, 0.36 mM Na_2_HPO_4_ x 2H_2_O, 5.5 mM glucose, 0.2 μM PGE1, pH 7.4), with the washing step repeated twice. Ultimately, the washed platelets were resuspended to a final concentration of 50–100 × 10^3^ platelets/µL using the washing buffer.

All subjects analyzed in this study had not taken any medication affecting platelet function for 10 days before venipuncture. Volunteers with any diseases of primary and secondary hemostasis or with acute illness at the time of venipuncture were excluded. Also, any sample with hemolysis and lipemia was discharged. The volunteers gave informed consent to participate in this study. The protocol was authorized by the ethics committee of the Universidad de Talca following the Declaration of Helsinki (approved by the 18th World Medical Assembly in Helsinki, Finland, 1964).

#### 2.2.2 Platelet aggregation assay

This assay was realized by light transmission, using a lumi-aggregometer (Chrono-Log, Havertown, PA, United States) (Recabarren-Leiva et al., 2021). Briefly, 408 µL of PRP (200–300 × 10^3^ platelets/µL) with a vehicle, AA (concentrations from 0 to 50 µM), or BPA (concentrations from 0 to 50 µM) were incubated and stirred for 5 min at 37°C before the addition of subthreshold concentrations of ADP or phorbol 12-myristate 13-acetate (PMA) (Hernández et al., 2019). As a positive control, ADP (4 µM) or PMA (200 nM) were used as agonists. We selected 50 µM as the maximum concentration since at that concentration it has been found to produce cellular toxicity. For example, acrylamide-induced lipid peroxidation (Salimi et al., 2021) and the BPA-inhibited growth of cyanobacterial cells (Yang et al., 2020).

In all conditions, the platelet aggregation was registered for 5 min and determined by AGGRO/LINK software (Chrono-Log, Havertown, PA, United States).

#### 2.2.3 Cytotoxic activity

Washed platelets (200–300 × 10^3^ platelets/µL) were incubated underthe same conditions mentioned above for 10 min at 37°C. After that, viability was determined by BD FACSLyric™ Clinical Flow Cytometry System (BD Biosciences, San Jose, CA, United States) using calcein-AM (Rywaniak et al., 2015). The supernatant was obtained by centrifugation at 900 *g* for 8 min and evaluated with a lactate dehydrogenase (LDH) cytotoxicity kit (Cayman Chemical, Ann Arbor, MI, United States) (Rywaniak et al., 2015). A solution of Triton X-100 was used as a positive control for each cytotoxicity assay.

#### 2.2.4 Reactive oxygen species (ROS) assay

ROS production was determined in WP (50 × 10^3^ platelets/µL). For this, platelets were incubated with 10 µM dihydroethidium (DHE) for 30 min at 37°C in a darkroom (Ramsey et al., 2015). Then, labeled platelets were incubated as previously described for 15 min at 37°C to increase ROS levels. ROS formation was analyzed by flow cytometry using a BD FACSLyric™ Clinical Flow Cytometry System (BD Biosciences, San Jose, CA, United States) (Walsh et al., 2014). Antimycin 10 μM was used as a positive control. The Antimycin is a specific inhibitor of complex III in the mitochondrial electron transport chain. This leads to superoxide (O_2_^.−^), hydrogen peroxide (H_2_O_2_) formationand other reactive oxygen species (ROS), contributing to oxidative stress (Park et al., 2007).

### 2.3 Statistical analysis

Platelet aggregation was assessed using blood samples obtained from six independent healthy volunteers, a sample size deemed appropriate based on the high reproducibility and low inter-individual variability of *ex vivo* platelet aggregation assays. Each volunteer served as their own control, with platelet responses measured before and after exposure to AA and BPA, allowing for a paired analysis to minimize variability and enhance statistical power. Platelets exhibit rapid and robust aggregation responses, and aggregation was quantified as a percentage of maximal aggregation, a well-established method that enables the detection of significant functional changes even with a moderate sample size. Data were analyzed using GraphPad Software version 6.0 (La Jolla, California, United States). Two or more measurements were performed for each test and expressed as mean ± standard error of the mean (SEM). Differences between groups were analyzed by Student’s t-test or ANOVA and p values <0.05 were considered significant.

## 3 Results

### 3.1 Bioinformatic analysis

#### 3.1.1 Identification of chemical-protein interaction of AA and BPA

**FIGURE 1:**
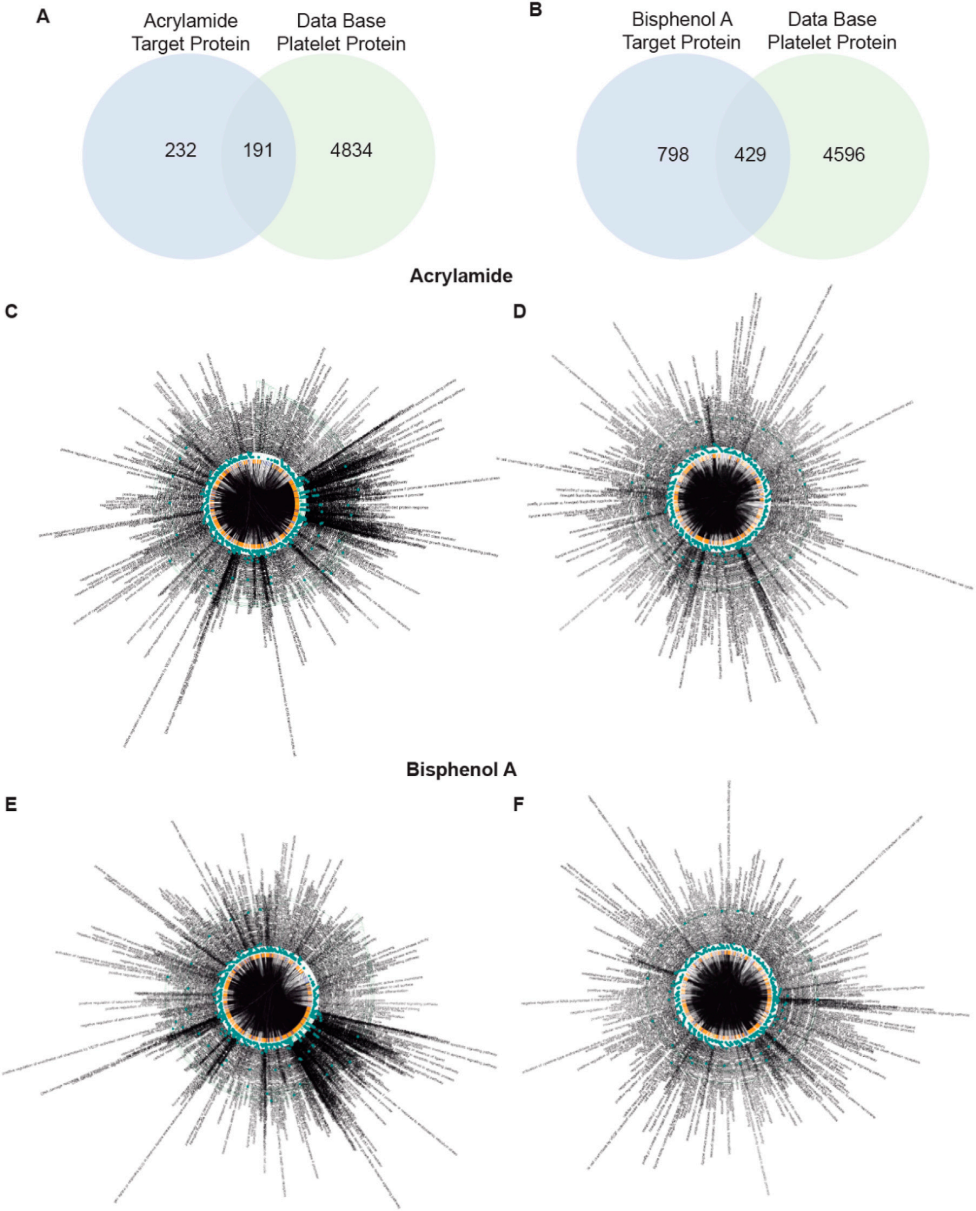
Screening of targets related to Acrylamide (AA) and Bisphenol A (BPA). **(A, B)** Venn diagram displays 191 and 429 overlapping genes between AA and BPA respectively, and platelet-related target genes. **(C–F)** A Biological Process network of targets. C, all target protein of AA; D, platelet target protein of AA; E, all target protein of BPA; and F, platelet target protein of BPA.

Of the total 423 proteins found to interact with AA, 191 were found in platelets (Figure 1A). In the case of BPA, a total of 1,227 proteins were detected, 429 of which were present in platelets (Figure 1B).

#### 3.1.2 GO and KEGG pathway analysis

GO, and KEGG pathway enrichment analyses were performed with two online platforms. The data showed that in the human body, 522 biological processes are affected by AA (Figure 1C) and 944 by BPA (Figure 1E). From these, we can highlight the regulation of pathways and gene expression related to apoptosis. This means that the intake of MP can profoundly affect fundamental functions in the human body. When we analyzed platelets, we found 322 and 602 biological processes affected by AA (Figure 1D) and BPA (Figure 1F), respectively. Apoptosis appeared again as one of the most relevant processes affected, in addition to oxidative stress. Therefore, the presence of either chemical present in food, beverages, and polluted air would affect fundamental processes in platelets.

**FIGURE 2:**
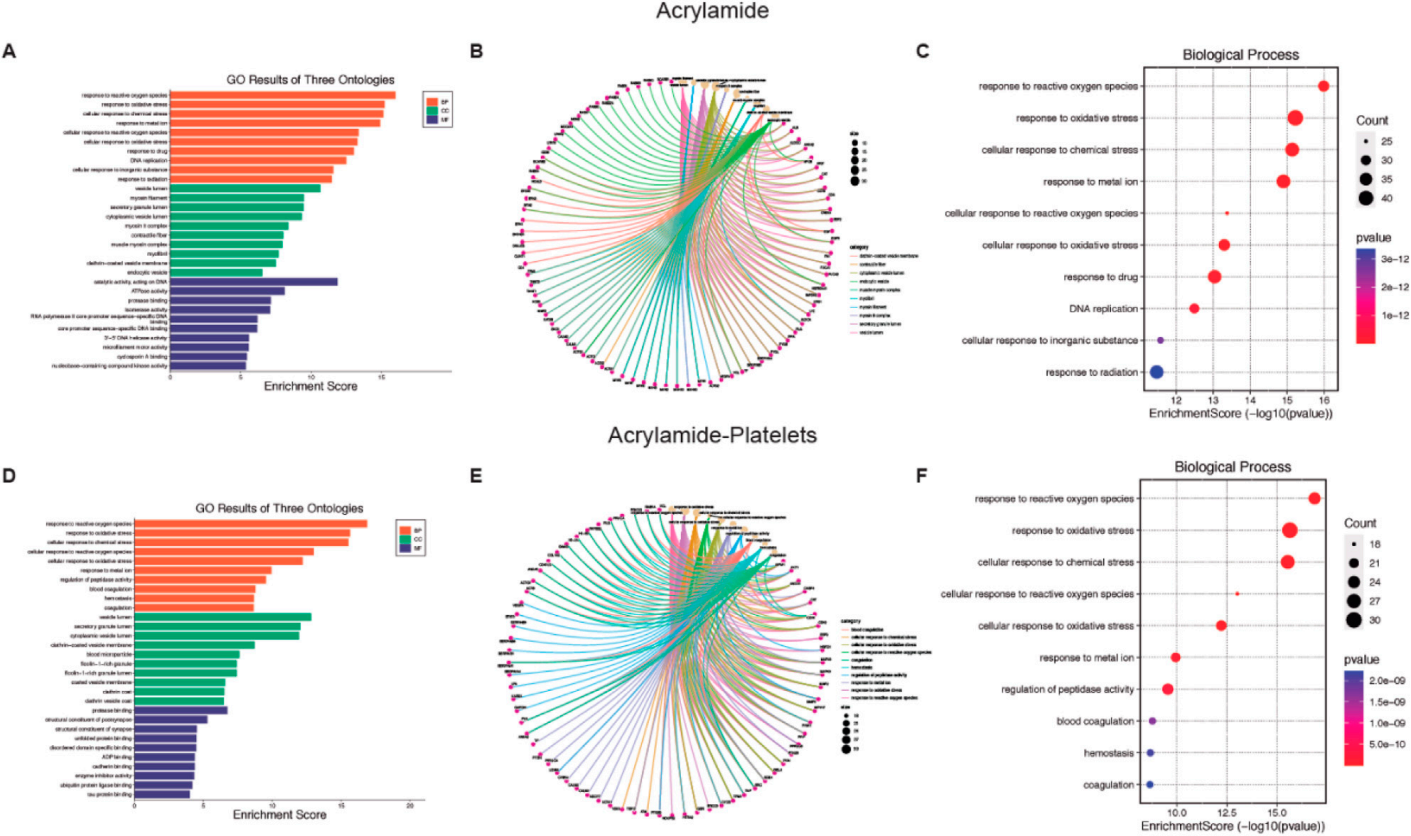
Biological function of Acrylamide (AA) and AA-platelet target protein. **(A, D)** Gene Ontology (GO) second class enrichment assay of AA and AA-platelets targets. **(B, E)** Cnetplot depicts the linkages of targets and the top ten biological concepts. **(C, F)** The bubble chart shows GO terms’ top 10 biological processes (BP).

**FIGURE 3:**
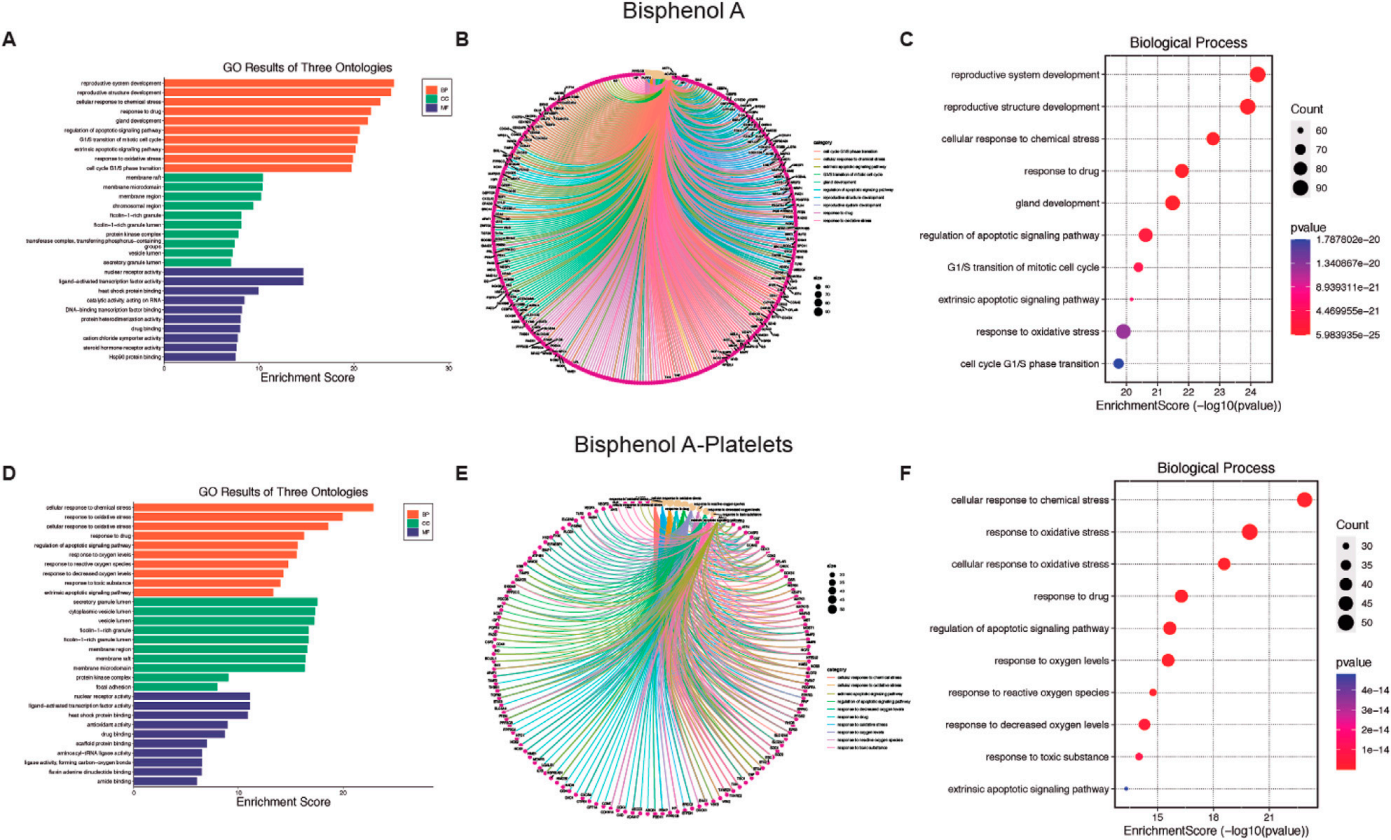
Biological function of Bisphenol A (BPA) and BPA-platelet target protein. **(A, D)** Gene Ontology (GO) second class enrichment assay of BPA and BPA-platelets targets. **(B, E)** Cnetplot depicts the linkages of targets and the top ten biological concepts. **(C, F)** The bubble chart shows GO terms’ top 10 biological processes (BP).

Given the large number of processes that affect these MP, we plotted the top 10 GO enrichment items (Figures 2, 3). Among the biological processes (BP) affected by AA, oxidative stress appears as one of the most relevant (Figure 2A). For cellular components (CC), see Supplementary Figures S1A–C for more details. Additionally, AA’s effects on molecular function (MF) are related to DNA and various enzymes such as isomerases (see Supplementary Figures S1D, F for more details). In the GO database, one gene may belong to multiple ontological terms. Therefore, plots (cnetplot and EnrichmentScoredotplot) visualizing size values and the relationships between genes and selected GO-BP terms were used to depict the data simply (Figures 2B,C).

In platelets, the BP associated with AA contamination is mainly related to oxidative stress (Figure 2D), the CC processes are related to intracytoplasmatic vesicles (see Supplementary Figures S2A-C for more details), and the MF homeostasis related to the regulation of proteins (see Supplementary Figures S2D–F for more details). As previously mentioned, the plots (cnetplot and EnrichmentScoredotplot) for the relationship between genes and selected GO-BP terms were generated (Figures 2E, F).

Concerning the BP affected by BPA, a relationship with the reproductive system (Figure 3A) arises, with the CC (see Supplementary Figures S3A–C for more details) present at the cell membrane, and MF was mainly transcription factors (see Supplementary Figures S3D–F for more details). Also, for BPA the cnetplot and EnrichmentScoredotplot were generated entirely (Figures 3B, C). Like AA, BPA affected oxidative stress processes in platelets (Figure 3D) through modifications in the CC at vesicles (see Supplementary Figures S4A–C for more details). Finally, transcription factors are the MF targeted by BPA (see Supplementary Figures S4D–F for more details) which in the case of platelets would be a non-genomic function. Also, cnetplot and EnrichmentScoredotplot were generated (Figures 3E, F).

**FIGURE 4:**
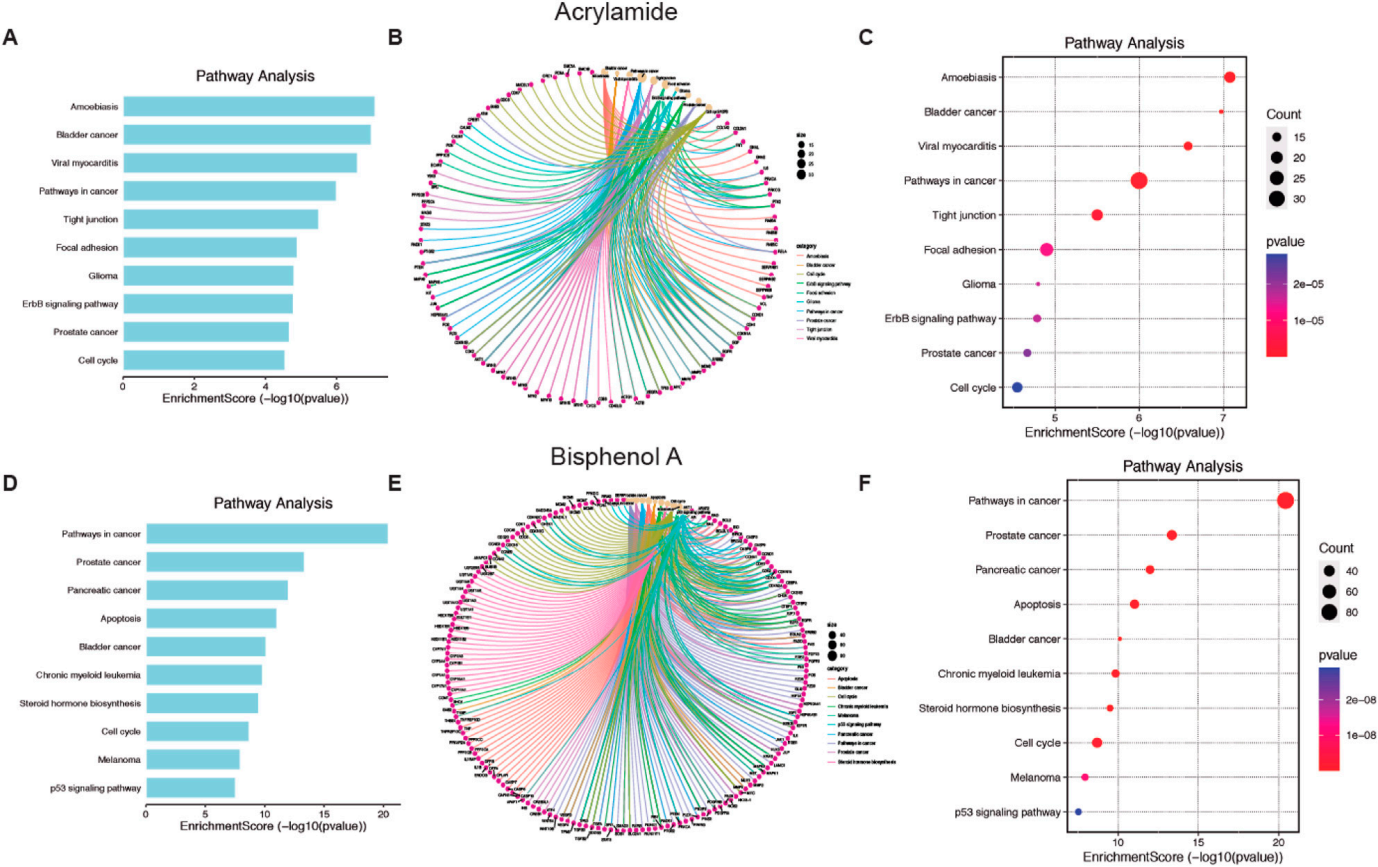
Pathways enrichment analysis of Acrylamide (AA) and Bisphenol A (BPA). **(A, D)** Bar chart showing the ten top AA and BPA pathways. **(B, E)** Cnetplot depicts the linkages of targets and the top ten pathways. **(C, F)** Bubble chart of top 10 signaling pathways linked to AA and BPA.

Finally, the top 10 KEGG pathway enrichment items are shown in Figures 4A, D for AA and BPA, respectively. The KEGG pathways enrichment analysis mainly includes a cancer pathway for both MP (Supplementary Figures S5A, B for AA and BPA, respectively). Also, the plots (cnetplot and EnrichmentScoredotplot) were generated (Figures 4B, C, E, F). Where it can be seen that both compounds, through the activation of different pathways such as MAPK or activation of p53, are related to different types of cancer such as Glioma, prostate, and cell cycle alteration.

#### 3.1.3 Screening of candidate targets and interaction network analysis of targets

**FIGURE 5:**
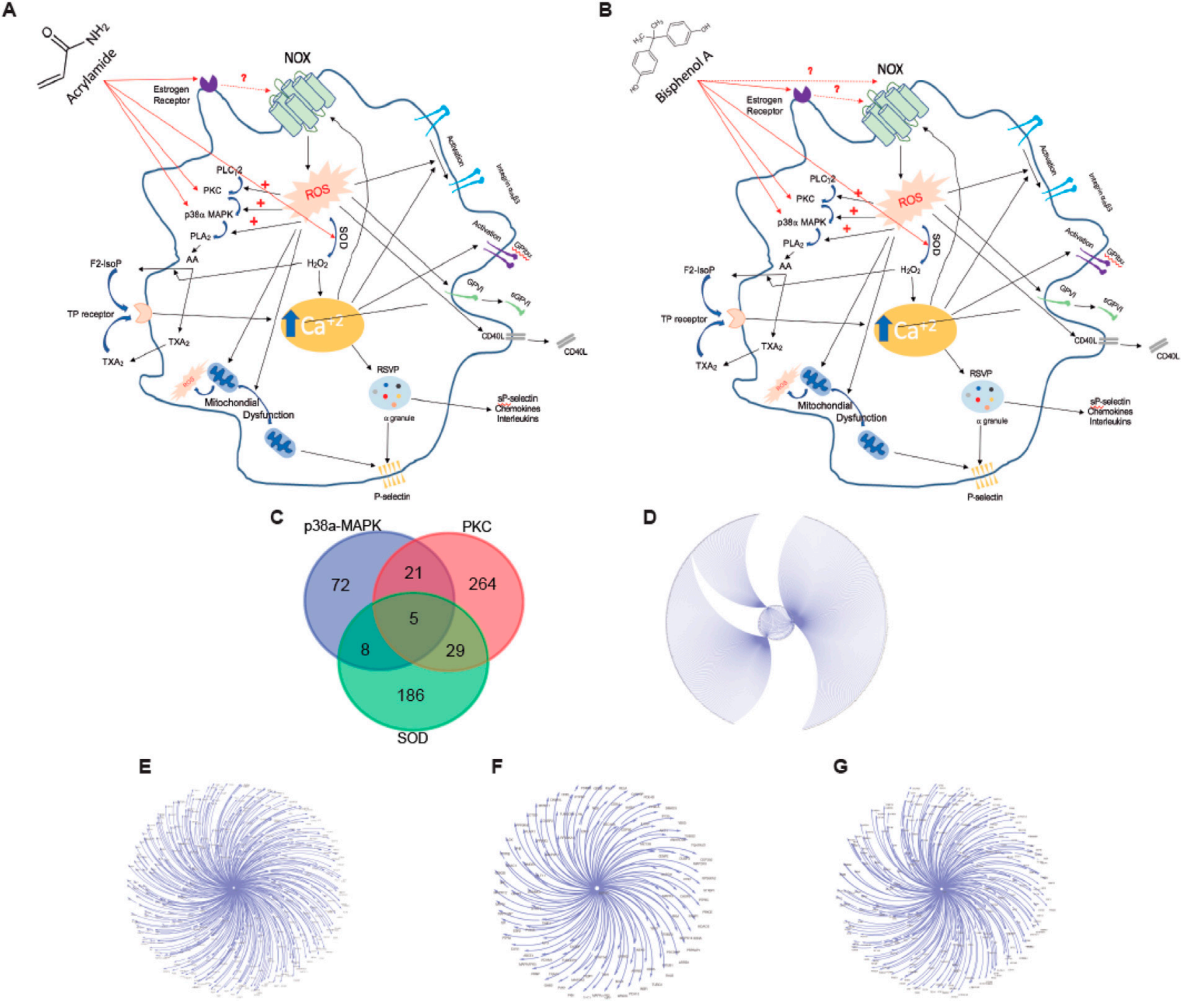
Platelet signaling pathways that trigger ROS production by **(A) **Acrylamide (AA) and **(B)** Bisphenol A (BPA). The diagram that shows the most important proteins that participate in the production of ROS by platelets also indicates the possible target proteins of AA and BPA. **(C)** Venn diagram displays overlapping platelet protein targets between three targets of AA and BPA. **(D–G)** Platelet protein association networks, these network interactions were generated with Cytoscape V3.4 (spring-embedded layout); the protein was represented by text (nodes), and the lines (edges) connecting the two texts signify an interaction between two proteins. **(D)**, all target protein; **(E)**, PKC-platelet target protein; **(F)**, P38a-MAPK-platelet target protein; and **(G)**, SOD-platelet target protein.

As previously indicated, the main BP affected by AA and BPA in platelets is oxidative stress. First, we generated a model comprising the proteins and pathways associated with oxidative stress in platelets (Figures 5A, B). ROS formation is a key element in platelet oxidative stress. Thus, we decided to evaluate the level at which ROS production can be modulated in platelets pre-incubated with AA and BPA. As shown in Figures 5A, B, the generation of ROS activates PKC, P38α-MAPK, and SOD, which are common for both AA and BPA. Therefore, we performed a protein-protein interaction (PPI) network analysis to determine the effect of activating these proteins in platelets.

According to four databases (BioGRID, IID, APID, and IntAct), 585 genes were found in the platelets to be associated with the three proteins (PKC: 319; P38α-MAPK: 106 and SOD: 228; Figure 5C); these interactions were constructed with the use of Cytoscape software (Figures 5D–G). As shown in the intersection by the Venn diagram, five common targets were identified (Figure 5C). Therefore, our data supports platelet activation by AA or BPA through the generation of ROS.

#### 3.1.4 Interaction prediction of AA and BPA with protein targets by molecular docking

As we determined above, the three common proteins activated by AA and BPA are PKC, P38α-MAPK, and SOD. Therefore, we performed a docking prediction to check the activation of these proteins by AA and BPA.

**FIGURE 6:**
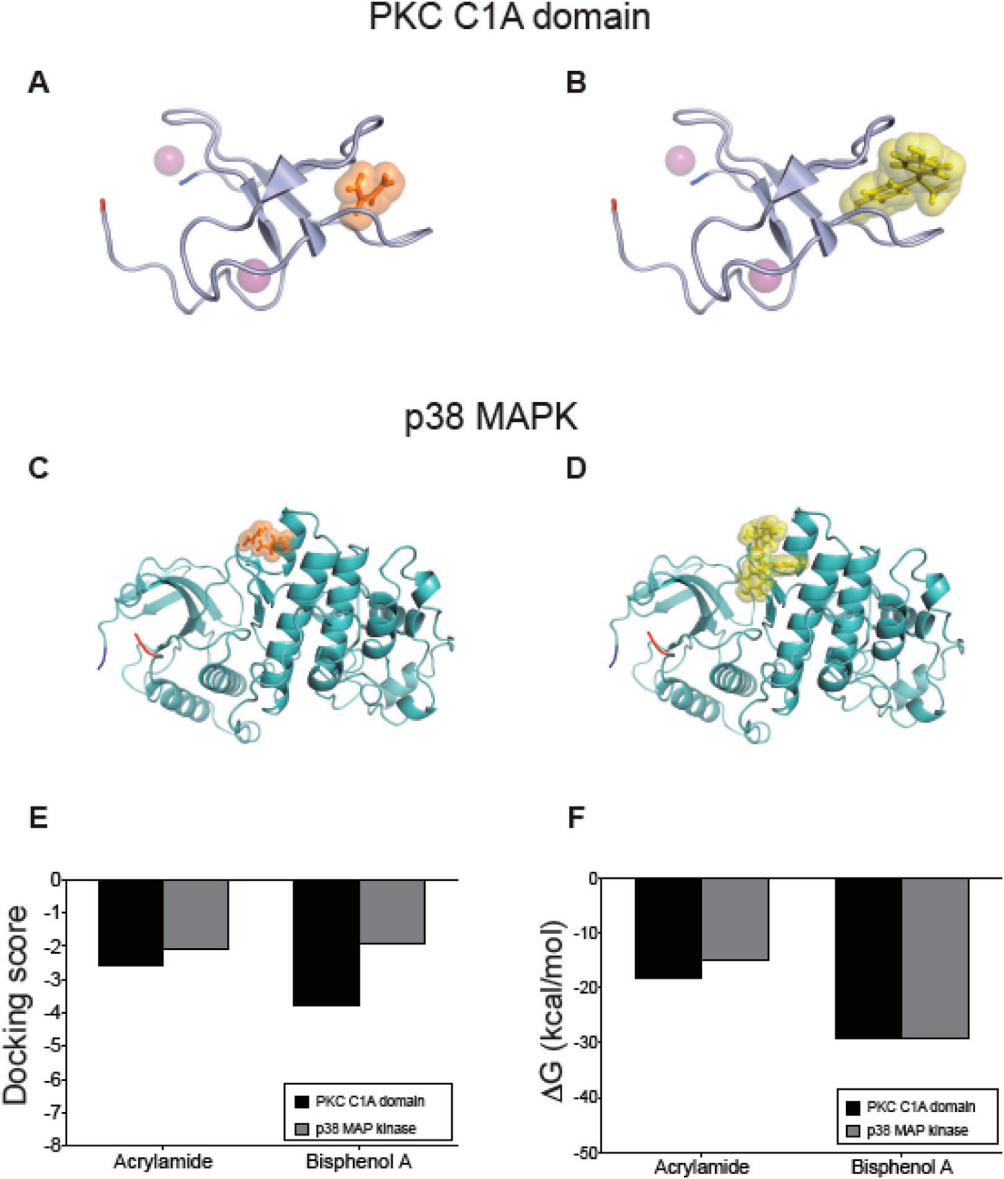
Interaction between acrylamide and bisphenol with kinases predicted by docking protein-ligand. Representative complexes were obtained by docking prediction using the structures of PKC C1A domain **(A, B)** and p38 MAPK **(C, D)**. AA and BPA show orange and yellow spheres, respectively. The N-terminal is marked in blue and the C-terminal in red. The purple spheres in PKC correspond to Zn atoms. The graphs summarize the **(E)** docking score values reported by Glide, and **(F)** the ΔG_bind_ values calculated using the MM-GBSA method using Prime. All figures were created using PyMOL.

In PKC, we analyzed the C1A and C1B domains that correspond to the diacylglycerol (DAG) binding site that stimulates its activity. Using the structures of each domain, we performed protein-ligand docking using phorbol 12-myristate 13-acetate (PMA), a potent PKC activator that binds to these regions, where the binding affinity values calculated by Vina software were −6.4 and −4.7 kcal/mol for C1A and C1B, respectively. Considering these values, the C1A domain was selected for a second docking prediction with AA and BPA using Glide (Figures 6A, B), which showed favorable interaction with docking score values of −2.587 and −3.806 (Figure 6E), accompanied by ΔG_bind_ of −18.12 kcal/mol and-29.04 kcal/mol (Figure 6F). A more detailed analysis of the interface protein-ligand shows the presence of amino acids ^180^VWGLNKQG^187^, and^166^FFP^168^ that define a mostly hydrophobic cavity, where ^185^K forms a hydrogen bond with both molecules, while ^182^G and ^167^F form a similar bond exclusively with AA (see Supplementary Figure S6 for more details).

The second target of the group of protein kinases was the MAP kinase p38, which after the first docking with Vina software, we confirmed that the interaction of AA and BPA occurs at the same binding site described for MKKB3, a p38 activator (Figures 6C, D). This allowed us to position an interaction grid for a more detailed evaluation performed with Glide, where AA and BPA interacted favorably with p38 MAP Kinase showing docking score values of −2.063 and −1.907, respectively (Figure 6E). These complexes were analyzed by calculating their ΔG_bind_, which reached −15.031 kcal/mol for AA, and −29.2 kcal/mol for BPA (Figure 6F), indicating that they are structurally and energetically favorable. The binding site for both molecules is slightly more polar than PKC and is formed by two groups of amino acids, including ^110^GA^111^, ^115^NI^116^, ^119^CQ^120^, ^158^VNE^160^, ^162^C, and ^125^DH^126^, ^129^F, ^161^DCE^163^, ^311^Y. The ^120^Q, ^125^D, and ^163^E are responsible for forming hydrogen bonds that contribute to the stabilization of the complexes (see Supplementary Figure S6 for more details). These results predict the interaction of both molecules with the PKC and p38 MAP Kinase (Figures 6E, F).

**FIGURE 7:**
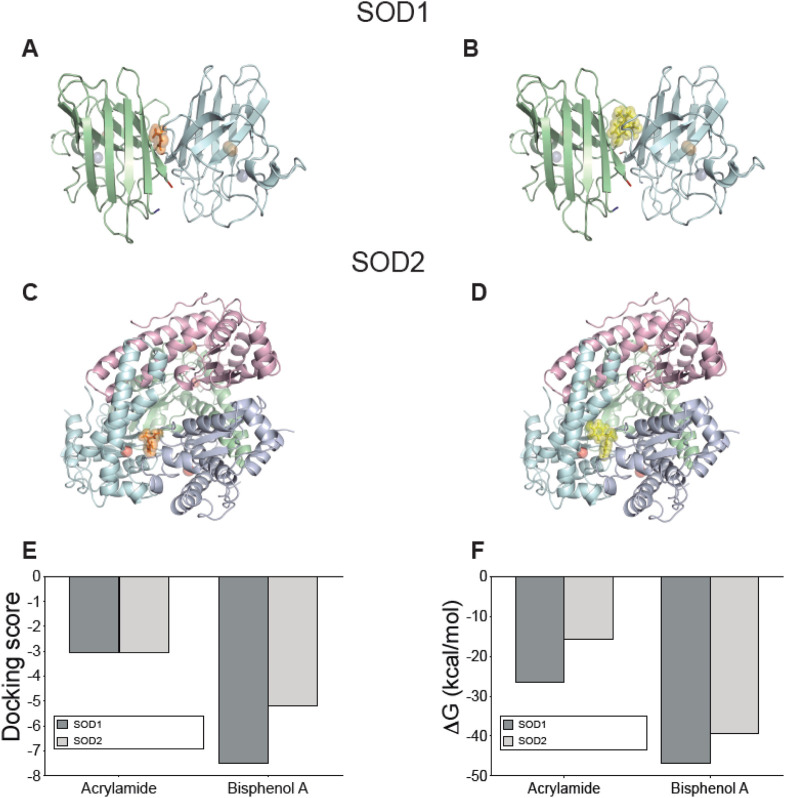
Interaction between acrylamide and bisphenol with Superoxide dismutase isoforms expressed on platelets predicted by docking protein-ligand. Representative complexes were obtained by docking prediction using the structures of SOD1 **(A, B)** and SOD2 **(C, D)**. AA and BPA show orange and yellow spheres, respectively. The N-terminal is marked in blue and the C-terminal in red. The blue spheres in SOD1 correspond to Zn atoms while the orange spheres are Cu atoms. Meanwhile, the red spheres of SOD2 are Mn atoms. The graphs summarize the **(E)** docking score values reported by Glide, and **(F)** the ΔG_bind_ values calculated using the MM-GBSA method using Prime. All figures were created using PyMOL.

Finally, we analyzed both SOD1 and SOD2, which are expressed in platelets. In the case of SOD1, the interaction with either AA or BPA was confirmed, and this occurred at the same binding site as ebselen, a selenorganic compound with antioxidant properties that stabilizes SOD1 (Figures 7A, B). SOD1’s structure is a dimer (chains A and F) where the binding site for the molecules is located intersubunit, in a mostly hydrophobic symmetrical region formed by amino acids 5VCV7, 9K, 51G, 53N, and 146CGVIG150 of each subunit (see Supplementary Figure S8 for more details). The interaction with SOD1 was stable and structurally and energetically favored, as shown by the docking score values of −3.046 and −7.508 for AA and BPA, respectively (Figure 7E), along with their respective ΔG_bind_ values of −26.56 kcal/mol and −46.79 kcal/mol, respectively (Figure 7F). Additionally, three hydrogen bonds with amino acids 148V of chain A, and 51G and 53N of chain F were detected for AA (see Supplementary Figure S8 for more details). Differences in the interface were found where BPA generates three hydrogen bonds involving amino acids 7V of chain F and 148V of both chains A and F, accompanied by a pi-cation bond with 9K of chain A (see Supplementary Figure S8 for more details).

The case of SOD2 is quite particular since its quaternary structure corresponds to a tetramer. Our predictions indicate an interaction at an intersubunit site (Figures 7C, D), and the docking results showed a docking score of −3.052 and −5.190 for AA and BPA, respectively (Figure 7E). Similarly, ΔG_bind_ calculations confirmed that the interaction is energetically favorable, reaching −15.72 kcal/mol for AA and −39.36 kcal/mol for BPA (Figure 7F). A detailed analysis of the interactions that stabilize the complexes with each molecule showed that, in the case of AA, it was possible to detect the formation of alternating hydrogen bonds with the amino acids ^118^V and ^120^G of the B/D chains or with the amino acids ^162^E and ^165^Y of chain D (see Supplementary Figure S9 for more details). For BPA, two hydrogen bonds were formed with amino acids ^67^N of chain B and ^162^E of chain D, along with a pi-pi stacking involving ^66^F of chain B and a pi-cation interaction with ^173^R of chain D (see Supplementary Figure S9 for more details).

Given the chemical nature of AA and BPA, it may be predicted that both MP have a high probability of interacting with multiple targets at discrete sites within their structures. Therefore, the four target proteins analyzed can form stable complexes with both molecules, in conserved binding sites with other activators described for each of them, which correlates with the effects described in this work.

#### 3.1.5 Ability to cross biological membranes of AA and BPA evaluated by MD simulations

**FIGURE 8:**
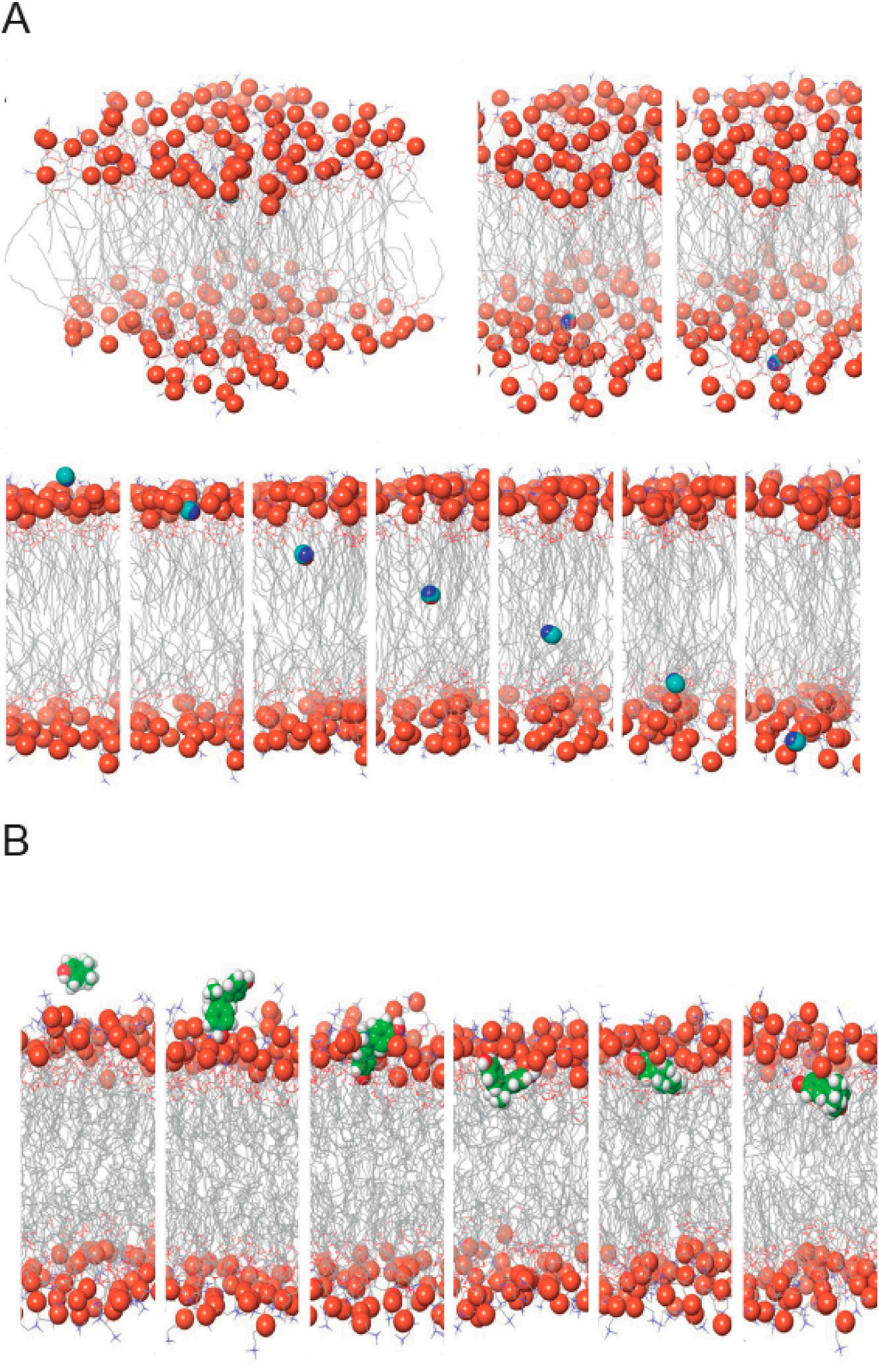
Molecular Dynamics Simulations of AA and BPA within a Phospholipid Bilayer. **(A)** Spatial Dynamics of AA within the Membrane Bilayer. The upper panel illustrates the interactions of AA with the external surface of the lipid membrane, revealing its association patterns. The lower panel showcases the translocation of AA as it traverses across the lipid bilayer. **(B)** Exploring Interactions of BPA within the Lipid Bilayer. This representation captures the dynamic displacements and insertion events of BPA within the phospholipid bilayer, shedding light on its behavior and influence on the membrane environment.

The results of the molecular dynamics simulations provide compelling evidence that both AA and BPA can cross the plasma membrane (Figure 8, Supplementary Videos S1, S2). The simulations were conducted to understand the permeation behavior of these molecules across the lipid bilayer of the plasma membrane. Our findings indicate that AA and BPA exhibit distinct modes of interaction with the lipid membrane, enabling their translocation from the extracellular aqueous phase to the intracellular environment. For AA, the simulations reveal that these molecules mainly interact and are concentrated on the outer membrane surface (Figure 8A). However, only a small fraction of AA successfully penetrates the lipid bilayer and enters the cytoplasm. This limited translocation may be attributed to the hydrophilic nature of AA, which prefers to remain on the membrane surface. On the other hand, BPA (Figure 8B) demonstrates contrasting behavior. A larger fraction of BPA compared to AA molecules is adsorbed onto the cell membrane and then effectively translocated into the cytoplasm through lipid-water partitioning. The hydrophobic characteristics of BPA contributed to its efficient membrane crossing and intracellular localization.

Our molecular dynamics simulations offer compelling evidence that both MP can cross the plasma membrane, albeit through distinct mechanisms. These findings shed light on their potential cytotoxicities and underscore the importance of understanding their interactions with functional biomacromolecules.

### 3.2 Validation and functional analysis of the effects of AA and BPA on platelets

**FIGURE 9:**
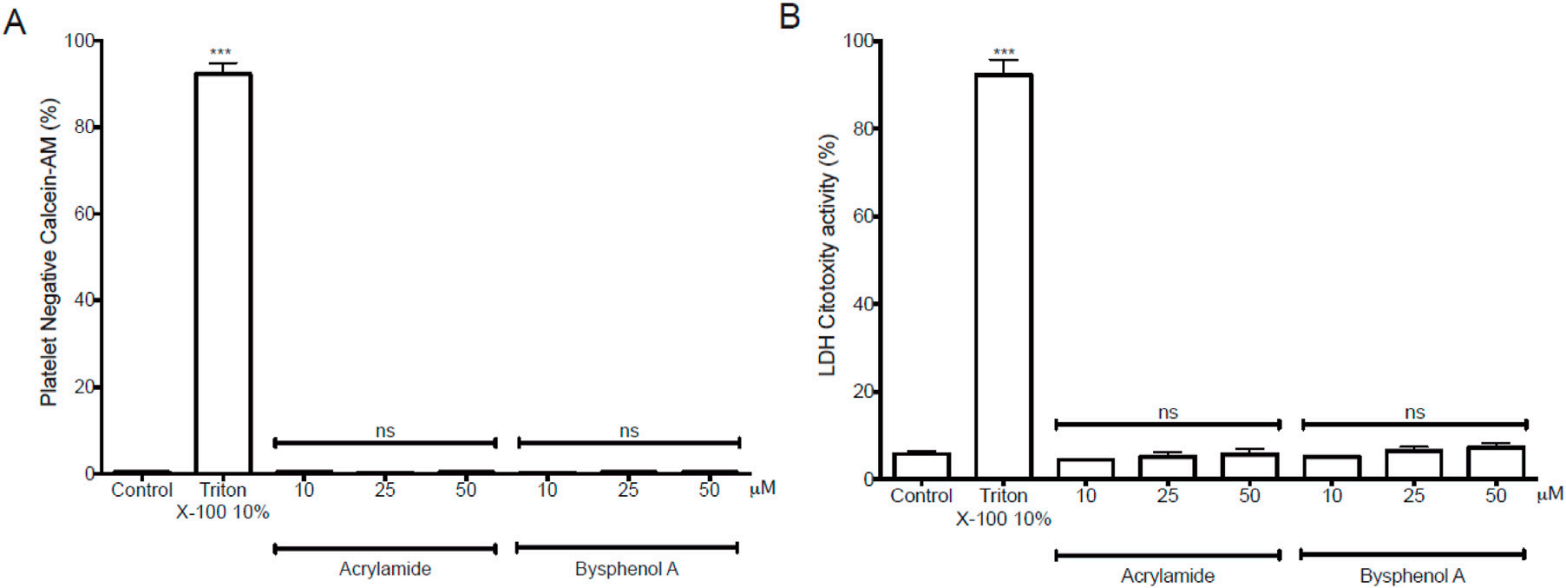
Cytotoxicity exposure induced by Acrylamide (AA) and Bisphenol A (BPA) in platelets. **(A)** Platelet viability using calcein-AM. The populations of calcein-negative platelets (anti-CD61) were non-viable cells. **(B)** LDH release from platelets was analyzed with the LDH cytotoxicity assay kit in the supernatant and measured at 490 nm in a microplate reader.

After the theoretical studies, we decided to confirm our results in cell studies. According to reports in the literature for cell assays with AA and BPA(Attoff et al., 2016; Gao et al., 2019; Karmakar et al., 2017; Sellier et al., 2015),we decided to work with MP concentrations of up to 50 µM.First, we determined the extent of cytotoxicity induced by MP on WP by performing calcein-AM and LDH release assays, as shown in Figure 9. Neither AA nor BPA significantly increased the calcein-negative population (cytotoxic effect) compared to the non-treated control group (Figure 9A). Similarly, cytotoxicity measured by LDH release showed similar results (Figure 9B). In both experiments, we included a positive control where WP was incubated with Triton X-100 at 10%. Thus, the positive control (93.1% ± 2.6% and 92.3% ± 3.4% for AA and BPA, respectively, in the calcein assay) was significantly higher compared to the basal control (0.3% ± 0.1% and 5.8% ± 0.7% for AA and BPA, respectively, in the LDH assay; p < 0.0001).

#### 3.2.1 Effect of AA and BPA on human platelet activation

**FIGURE 10:**
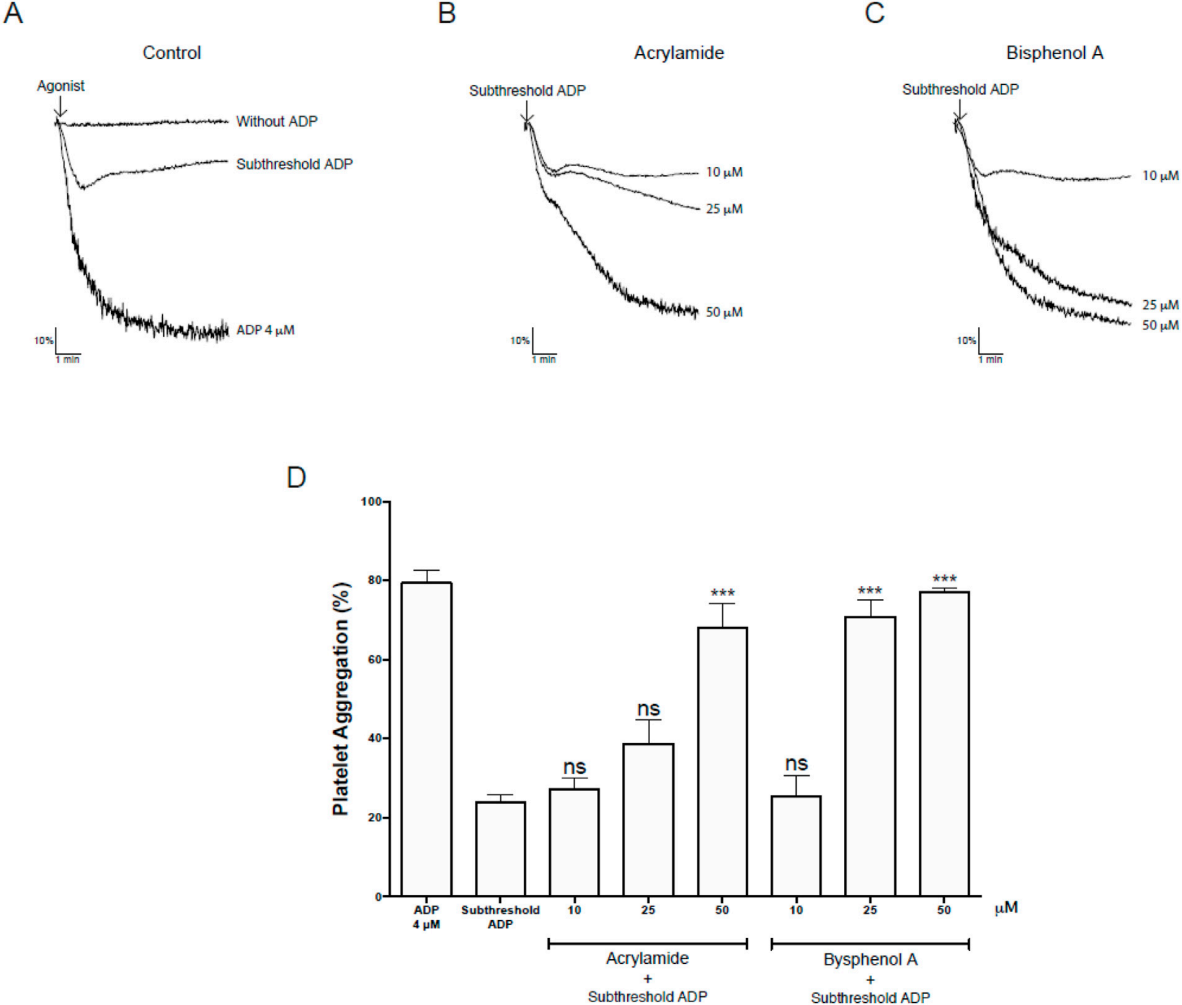
Effects on platelet aggregation mediated by Acrylamide (AA) and Bisphenol A (BPA) in platelets. **(A–C)** Upper panel: representative kinetic aggregation obtained for each condition. **(D)** the graph summarizes the percentage of platelet aggregation. The results were presented from six independent volunteers (each donor executed as single triplicates) and expressed as mean ± SEM.

Bioinformatic analysis showed that AA and BPA promote platelet activation through oxidative stress (Figures 5A, B). Since AA and BPA did not exert cytotoxicity in platelets, we investigated whether the MP could influence platelet function by assessing platelet aggregation. Platelets pre-incubated with AA or BPA (0, 10, 25 and 50 µM) and stimulated with a subthreshold concentration of ADP, the aggregation induced by the agonist (23.9% ± 1.9%) was enhanced in the presence of MP (68.0% ± 6.0% and 70.8% ± 4.2% for 50 µM AA and 25 µM BPA, respectively; Figures 10A–D). These results were similar to those observed with 4 µM ADP (79.3% ± 3.2%), in the absence of MP (Figures 10A, D). In the absence of aggregation agonists, AA nor BPA did not cause any platelet aggregation even at the highest concentration tested (see Supplementary Figure S10 for more details). Overall, these results indicate that the platelet aggregation was increased by AA or BPA.

#### 3.2.2 Effect of AA and BPA on the production of intraplatelet ROS

Our data show that AA and BPA produce platelet activation (Figure 10), and bioinformatic analyses indicate that one of the possible mechanisms could be oxidative stress (ROS; Figures 2D–F; Figures 3D–F). Therefore, we proceeded to measure intraplatelet ROS levels. Considering the preceding results, we evaluated the effects of AA and BPA on ROS formation at concentrations of up to 50 μM and 25 μM, respectively.

**FIGURE 11:**
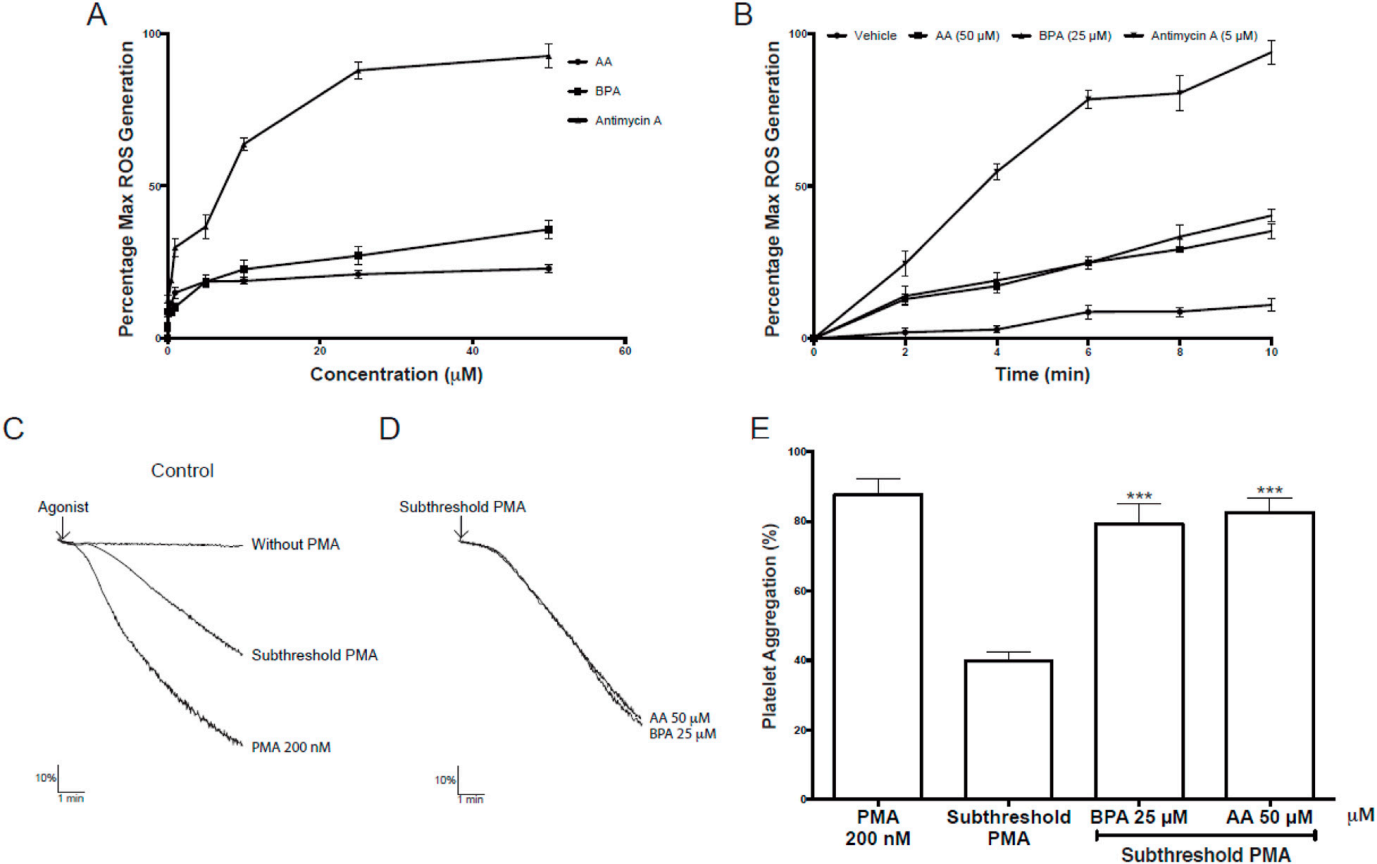
Effects of Acrylamide (AA) and Bisphenol A (BPA) on platelets. **(A, B)** Intraplatelet reactive oxygen species (ROS) generation was measured using a DHE probe in a flow cytometer. The platelets were identified as the CD61^+^ population and were analyzed in terms of change in mean fluorescence intensity (ROS production). **(C–D)** representative kinetic aggregation obtained for each condition. **(E)** the graph summarizes the percentage of platelet aggregation; PMA was used as a platelet agonist. The results were presented from six independent volunteers (each donor executed as single triplicates) and expressed as mean ± SEM.

Figure 11A shows the effect of AA or BPA on the production of platelet ROS, quantified by the fluorescence of the DHE probe. A significant concentration-dependent increase in ROS production was observed in platelets treated with AA, BPA, and antimycin A reaching maximal effects at 50 μM AA and 25 μM BPA (35.2% ± 2.3% and 40.3% ± 2.0%, respectively). The increase in ROS production was also time-dependent (Figure 11B), suggesting that both AA and BPA are exerting their effects by entering the platelet and activating intracellular pathways.

#### 3.2.3 Effect of AA and BPA on the activation of intraplatelet PKC

PMA is known to activate PKC and thereby cause cell growth arrest, apoptosis, or cell activation via signal transduction (Tahara et al., 2009). As for ROS, our data showed that AA and BPA can induce platelet activation through the activation of PKC protein (Figure 5). Therefore, we evaluated PKC activation in platelet aggregation using PMA as an agonist to determine if AA and BPA can enhance PKC effects.

Figures 11C–E, show the effect of AA or BPA on the activation of platelet PKC. The stimulation with 200 nM PMA induced platelet aggregation to 87.8% ± 4.6%, demonstrating platelet functionality in these assays (Figures 11C, E). Then, the platelets were pre-incubated with AA or BPA (50 and 25 μM, respectively) and stimulated with a subthreshold concentration of PMA which showed a 50% decrease in total aggregation compared to 200 nM PMA (Figures 11C, E). Importantly, when platelets were preincubated with AA or BPA and then stimulated with a subthreshold concentration of PMA, a significant increase in platelet aggregation was observed (82.5% ± 4.1% and 79.3% ± 5.8% for 50 µM AA and 25 µM BPA, respectively). In both cases, the enhancement was twice the observed for the subthreshold PMA concentration (39.5% ± 2.6%; Figures 11C, E).

## 4 Discussion

This is the first study to demonstrate the correlation between plastic toxic chemicals (AA and BPA) and platelet activation. This means that chronic exposure of humans to these harmful substances caused by the current high level of global plastic pollution will have detrimental effects on our health. Our results reveal that platelet activation produced by the increase in oxidative stress in the platelets occurs when they are in contact with these substances.

### 4.1 Biological implications of AA and BPA

In the initial steps of their actions, the differential transmembrane transport mechanisms of AA and BPA have significant implications for their biological effects. AA accumulation on the membrane surface may induce acute and indirect toxicities, leading to malnutrition and axial malformation in developing cells. Conversely, BPA’s ability to reach the cytoplasm could result in chemical damage to cells, potentially causing adverse effects like developmental disorders of the nervous system, cardiovascular complications, and obesity, among others (Naomi et al., 2022; Olsvik et al., 2020). The observed synergistic effects of the AA-BPA mixture further underscore the intricate interplay between these chemicals. The simulation-based findings align with previous studies that have elucidated the potential toxicities of both AA and BPA(Chen et al., 2016; Fei et al., 2010; Scaramella et al., 2022). Our results underscore the importance of considering distinct modes of interaction when evaluating their cytotoxic effects and developmental impacts.

### 4.2 AA and BPA as CVD risk factors

CVD is one of the most prevalent diseases worldwide. Its risk factors are very well known, such as family history, obesity, high blood pressure, dyslipidemia, and diabetes mellitus, among others (Hajar, 2017). However, it should be noted that a large number of patients with CVD do not have any of the risk factors mentioned above, and their cause is determined to be unknown (Bhatnagar, 2004; Khot et al., 2003). It has been determined that a new emerging factor that may play a role in the onset or increase of CVD is environmental contaminants (Bhatnagar, 2004), given that they can trigger cardiovascular toxicity. This suggests that exposure to different environmental contaminants such as MP could be part of the novel mechanisms. As an example, AA can form adducts with human hemoglobin in red blood cells, and these levels increase in smokers, thereby adding risk factors to the development of CVD (Pedersen et al., 2022). Most of the epidemiological studies that try to associate different toxic elements with CVD come from NHANES, where they have found a strong correlation between AA and BPA; therefore they could be considered as new CVD risk factors (Lang et al., 2008; Melzer et al., 2010; Neil et al., 2012; Zhang et al., 2018).

Platelets are crucial actors of CVD and play a critical role in hemostasis by maintaining the integrity of blood vessels. One of its essential functions is that when faced with damage to the endothelial tissue of the blood vessels, they are activated to repair the damaged tissue. However, unregulated platelet activation can trigger thrombosis, such as stroke. In addition, platelets are also involved in the development of another pathology such as atherosclerosis, which is also subsequently a trigger for thrombosis (Schulz and Massberg, 2012; Wagner and Burger, 2003).

### 4.3 Bioinformatic analysis

First, we used several bioinformatic tools (i.e., Interaction Networks databases, PlateletWeb, Pathway enrichment analysis tools), to verify our hypothesis that MP produces platelet activation and thus stimulates a prothrombotic state. The chemical databases we used are an excellent source for identifying the potential target proteins of these compounds, while the platelet database provided a novel systems biology workbench to analyze platelet proteins in the context of integrated networks. We found that both AA and BPA can modify many biological processes in the human body (522 and 944, respectively), many of which are in platelets (322 and 602, respectively). Our data confirmed the high level of toxicity of these compounds in the body and platelets (Figures 1C–F). By determining the specific processes affected in the body, we found that AA is associated with oxidative stress and BPA with alteration of the reproductive system (Figures 2, 3). We also confirmed that both chemicals are mainly associated with cancer (Figure 4).

### 4.4 AA and BPA increase oxidative stress

Many existing studies in the literature have established that the main mechanism of AA is to increase oxidative stress (Yousef and El-Demerdash, 2006; Zhao et al., 2017; Zhu et al., 2008). At the brain level, one of the proposed mechanisms suggests the decrease of glutathione (GSH), increasing ROS levels, increasing oxidative stress, and triggering neurodegeneration (Faria et al., 2019). Moreover, it has also been reported to increase oxidative stress in erythrocytes producing hemolysis, lipid peroxidation, and reducing GSH levels (Catalgol et al., 2009). It has been possible to determine the cellular mechanisms of AA in different cell lines. For example, in PC12 cells increase ROS levels and decrease GSH levels. It also increases the secretion of pro-inflammatory cytokines (tumor necrosis factor-α (TNF-α) and interleukin 6 (IL-6)), and also activates different pro-inflammatory signal transduction pathways and mitogen-activated protein kinases (MAPKs)) (Pan et al., 2018). In this sense, Pang et al. (2019), using HT-22 cells (Mouse Hippocampal Neuronal Cell Line), demonstrated that afterexposure to 100 μM BPAfor 6 h, exhibited stronger intracellular ROS levels. Also, Wang C. et al. (Wang et al., 2021), using SK-N-SH cells (Human Neuroblastoma Cell Line), found the same effect of increasing ROS levels in these cells. It showed that the effect could even be seen after 3 h and with concentrations <100 μM BPA. On the other hand, Hung et al. (2021), demonstrated that when AA was pre-incubated with THP-1 cells (Human Monocytic Cell Line), there was a considerable increase in ROS before 30 min, and that this increase is sustained until 4 h. Similar results were obtained in cells derived from a glioma and astrocytes where it was demonstrated that AA produced an increase and accumulation in ROS and also generated mitochondrial dysfunction (Deng et al., 2022; Yang et al., 2021).

In splenocytes, it was shown that AA increases cell apoptosis by stimulating the activation of caspases 8 and 9. At the mitochondria level, it activates complexes I and III of the electron transport chain, increasing ROS levels (Zamani et al., 2017); it even produces increased damage to genomic (Mori et al., 2022) and mitochondrial DNA (Yang et al., 2021). These data follow the results presented in the current work, being the modification of ROS production and the increase of oxidative stress one of the most important mechanisms of the harmful effects of AA.

There is also abundant literature associating BPA levels with damage to the reproductive system. It triggers several pathologies such as decreased fertility in males and follicle loss in females, cryptorchidism, testicular dysgenesis syndrome, and cancer (Yang et al., 2021). BPA is a xenoestrogen, which can both mimic the action of estrogen and bind to the nuclear estrogen receptors, ERα, and ERβ (Wisniewski et al., 2015). It has adverse effects on fertility, delays the onset of female puberty, and influencesthe estrous cycle (Wisniewski et al., 2015). Also, in male subjects, interference with stimulating follicle hormone inhibits testosterone, estradiol levels, and sexual function. Concerning sperm reduced motility, sperm DNA damage, normal morphology, sperm concentration, and at molecular levels, altered epigenetic pattern (De Toni et al., 2020). Among the molecular mechanisms, the increase in oxidative stress is relevant, considerably raising ROS levels which directly damage the sperm (Aitken et al., 1991). ROS has also been shown to damage lipids, proteins, and DNA (Aitken et al., 1995). At the membrane level, it has been reported that BPA activates estrogen and G protein-coupled (GPR30) receptors. In addition, through ROS formation, activation of several cytoplasmic proteins has been reported, such as phosphatidylinositol 3-kinase (PI3K), mitogen-activated protein kinase (MAPK), PKC, protein kinase A (PKA), and p38 MAP kinases (Nadal et al., 2000). One of the main mechanisms forMP is the increasing in oxidative stress, where ROS is the main component.

In this sense, our bioinformatic approach showed that exposing platelets to AA and BPA would considerably increase oxidative stress. In addition, it has been shown that platelets are capable of increasing the production of intraplatelet ROS when faced with a stimulus; later, as a consequence of this increase, platelet activation and increased prothrombotic state or thrombosis would occur (Nadal et al., 2000; Qiao et al., 2018). The increased production of intraplatelet ROS, in turn, alters mitochondria function and boosts platelet activation in an auto-amplifying loop (generating a platelet phenotype: pro-activatory, pro-adhesive, pro-aggregatory, and pro-thrombotic) (Iuliano et al., 1997).

Abundant literature shows the activation of different proteins (i.e., PKC (Tan et al., 2013; Triningsih et al., 2021), p38 (Lee et al., 2008; Lee et al., 2014), and SOD (Kabuto et al., 2003; Mallepogu et al., 2013)) supports the effect of the possible interaction between AA and BPA and platelets. Our work demonstrates and confirms the specific interaction between AA and BPA with these proteins at the activation site of each one of them, which translates into the aforementioned effect. It is essential to highlight that for all the proposed models, the interaction between the two compounds and the three proteins was highly stable and structurally and energetically favored. These interactions were possible due to the formation of alternating hydrogen bonds with the amino acids of the different proteins; in this way, these interactions stabilized the different complexes. It has been determined that an excessive increase in oxidative stress can modify platelet function and lead to toxicity, resulting in excessive platelet apoptosis (Masselli et al., 2020). Therefore, we determined the cytotoxicity of these compounds and found that concentrations <50 μM did not generate cytotoxicity.

### 4.5 AA and BPA increase platelet aggregation

The incubation of platelets with different AA or BPA concentrations did not cause any platelet aggregation on their own even at the highest concentration tested. Nevertheless, our results showed a significant increase in platelet aggregation in the presence of MP by stimulating the aggregation induced by subthreshold agonist concentrations. This means both AA and BPA decrease the concentration of agonist needed to induce platelet aggregation thus prompting activation of platelets that could favor different pathologies such as atherothrombosis. In this way, different studies have shown that various platelet agonists at subthreshold concentrations elicit synergistic interactions and lead to aggregation by previously adding another platelet agonist in similar subthreshold concentrations (Hernández et al., 2019; Shah et al., 2001). The molecular mechanisms of these synergisms are intricate as the agonists used in the current study stimulated diverse intracellular responses.

### 4.6 AA and BPA increase intraplatelet ROS

ROS represents essential secondary messengers in signal transduction cascades and has been described as a critical step in platelet activation (Jang et al., 2014; Walsh et al., 2014). Furthermore, platelets are both the source and target of ROS(Lu et al., 2019; Tomasiak et al., 2004). Therefore, our results show that the generation of ROS by AA and BPA in platelets is concentration and time-dependent. Therefore, it would be an essential mechanism for platelet activation. We also show that one of the effects of putting the platelet in contact with these chemicals is the activation of PKC. The PKC is a significant regulator of platelet granule secretion, integrin activation, aggregation, spreading, and procoagulant activity; therefore it is vital in platelet activation and thrombus formation, both *in vitro* and *in vivo* (Harper and Poole, 2010). The activation of PKC by AA or BPA can be ascribed as a relevant mechanism of damage induced in platelets by these chemicals.

### 4.7 Limitations

While this study provides valuable insights into the effects of acrylamide (AA) and bisphenol A (BPA) on platelet function, several limitations should be considered when interpreting our findings. First, our study was conducted using *in vitro* models, which, while informative, may not fully capture the complexity of *in vivo* physiological conditions. Further validation using animal models or clinical studies is necessary to confirm the relevance of these molecular interactions in a physiological setting. Second, our bioinformatics approach identified key platelet-associated proteins affected by AA and BPA; however, potential confounding factors in the analysis should be acknowledged. While computational and mechanistic insights provide strong evidence of interaction, additional experimental validation is needed to confirm direct effects on platelet activation and aggregation. Lastly, although the concentrations of AA and BPA used in our study align with environmental exposure levels, variability in human metabolism, chronic exposure effects, and cumulative interactions with other environmental factors must be considered. Individual differences in exposure and detoxification pathways may influence the actual impact of these pollutants on cardiovascular health.

Despite these limitations, our study highlights important mechanistic insights into the role of AA and BPA in platelet function and provides a foundation for future investigations into their potential cardiovascular risks. Further research, particularly *in vivo* and epidemiological studies, will be essential to fully elucidate the clinical implications of our findings.

## 5 Conclusion

The continuous exposure (i.e., ingestion via food or water, inhalation, or dermal contact) to BPA and AA could result in a persistent exposure of platelets to contaminants. This sustained exposure may induce a state of pre-activation in platelets, predisposing them to aggregate and form thrombi at lower concentrations of platelet agonists. Consequently, the accumulation of circulating or stored AA and BPA in diverse tissues may foster an environment more susceptible to inadvertent platelet aggregation, thereby heightening the risk of experiencing undesired thrombotic events and elevating the likelihood of developing CVD.

## Data Availability

The original contributions presented in the study are included in the article/Supplementary Material. The raw data supporting the conclusions of this article will be made available by the authors, without undue reservation.
