# Supplementary material for: Acrylamide and bisphenol A: two plastic additives increase platelet activation, via oxidative stress
Source: Front Pharmacol. 2025 Apr 30;16:1526374. doi: 10.3389/fphar.2025.1526374 (PMC12075958; doi:10.3389/fphar.2025.1526374)
Supplement: Supplementary file 1 [file DataSheet1.zip › Supplementary Figures/Captions.DOCX]

**Supplementary Figure 1. Cellular Component and Molecular Function of Acrylamide (AA) target protein.** (A and D) Gene Ontology (GO) second class enrichment assay of AA targets. (B and E) Cnetplot depicts the linkages of the targets' top ten Cellular Component and top ten Molecular Functions. (C and F) Bubble chart showing the top 10 Cellular Component and top 10 Molecular Functions of GO terms.

**Supplementary Figure 2. Cellular Component and Molecular Function of Acrylamide (AA)-platelets target protein.** (A and D) Gene Ontology (GO) second class enrichment assay of AA-platelet targets. (B and E) Cnetplot depicts the linkages of the targets' top ten Cellular Component and top ten Molecular Functions. (C and F) Bubble chart showing the top 10 Cellular Component and top 10 Molecular Functions of GO terms.

**Supplementary Figure 3. Cellular Component and Molecular Function of Bisphenol A (BPA) target protein.** (A and D) Gene Ontology (GO) second class enrichment assay of BPA targets. (B and E) Cnetplot depicts the linkages of the targets' top ten Cellular Component and top ten Molecular Functions. (C and F) Bubble chart showing the top 10 Cellular Component and top 10 Molecular Functions of GO terms.

**Supplementary Figure 4. Cellular Component and Molecular Function of Bisphenol A (BPA)-platelets target protein.** (A and D) Gene Ontology (GO) second class enrichment assay of BPA-platelets targets. (B and E) Cnetplot depicts the linkages of the targets' top ten Cellular Component and top ten Molecular Functions. (C and F) Bubble chart showing the top 10 Cellular Component and top 10 Molecular Functions of GO terms.

**Supplementary Figure 5. Diagram of Cancer Signaling Pathways.** The pathway was exported from the KEGG pathway (KEGG reference: hsa05200 for Acrylamide and Bisphenol A) as a diagram. Black arrows symbolize direct molecular interaction or relation between molecules (white squares) or other types of molecules or chemical compounds (unfilled circles), a +p means phosphorylation or –p dephosphorylation, while black arrows with dashed lines denote an indirect effect. The target genes are mapped in green.

**Supplementary Figure 6. Binding site analysis of PKC C1A domain for acrylamide and bisphenol predicted by docking protein-ligand.** A detailed analysis of the interface of the complex showing the amino acids involved and its corresponding Ligand Interaction Diagram between PKC C1A domain with AA (A and B) or BPA (C and D). AA and BPA show orange and yellow spheres, respectively. Labeled amino acids are located less than 4Å away from the ligand. Ligand Interaction Diagrams were created by Maestro. All images were generated with PyMOL.

**Supplementary Figure 7. Binding site analysis of p38 MAPK for acrylamide and bisphenol predicted by docking protein-ligand.** A detailed analysis of the most representative complexes interface shows the amino acids involved and its corresponding Ligand Interaction Diagram between p38 MAPK with AA (A to D) or BPA (E to H). AA and BPA show orange and yellow spheres, respectively. Labeled amino acids are located less than 4Å away from the ligand. Ligand Interaction Diagrams were created by Maestro. All images were generated with PyMOL.

**Supplementary Figure 8. Binding site analysis of SOD1 for acrylamide and bisphenol predicted by docking protein-ligand.** A detailed analysis of the interface of the complex showing the amino acids involved and its corresponding Ligand Interaction Diagram between SOD1 with AA (A and B) or BPA (C and D). AA and BPA show orange and yellow colored spheres, respectively. The dimer-forming chains are cyan and green. Labeled amino acids are located less than 4Å away from the ligand. Ligand Interaction Diagrams were created by Maestro, and all images were generated with PyMOL.

**Supplementary Figure 9. Binding site analysis of SOD2 for acrylamide and bisphenol predicted by docking protein-ligand.** A detailed analysis of the most representative complexes interface shows the amino acids involved and its corresponding Ligand Interaction Diagram between SOD2 with AA (A and F) or BPA (G to H). AA and BPA show orange and yellow spheres, respectively. The tetramer-forming chains in cyan, green, pink, and blue. Labeled amino acids are located less than 4Å away from the ligand. Ligand Interaction Diagrams were created by Maestro, and all images were generated with PyMOL.

**Supplementary Figure 10. Effect of Acrylamide (AA) and Bisphenol A (BPA) on platelet aggregation.** (A) representative kinetic aggregation of conditions control. (B and C) incubation of different concentrations of AA and BPA with platelets. The results were presented from 6 independent volunteers (each donor executed as single triplicates) and expressed as mean ± SEM.
