# Supplementary material for: Acrylamide and bisphenol A: two plastic additives increase platelet activation, via oxidative stress
Source: Front Pharmacol. 2025 Apr 30;16:1526374. doi: 10.3389/fphar.2025.1526374 (PMC12075958; doi:10.3389/fphar.2025.1526374)
Supplement: Supplementary file 1 [file DataSheet1.zip › Supplementary Figures/Figure S7.PDF]

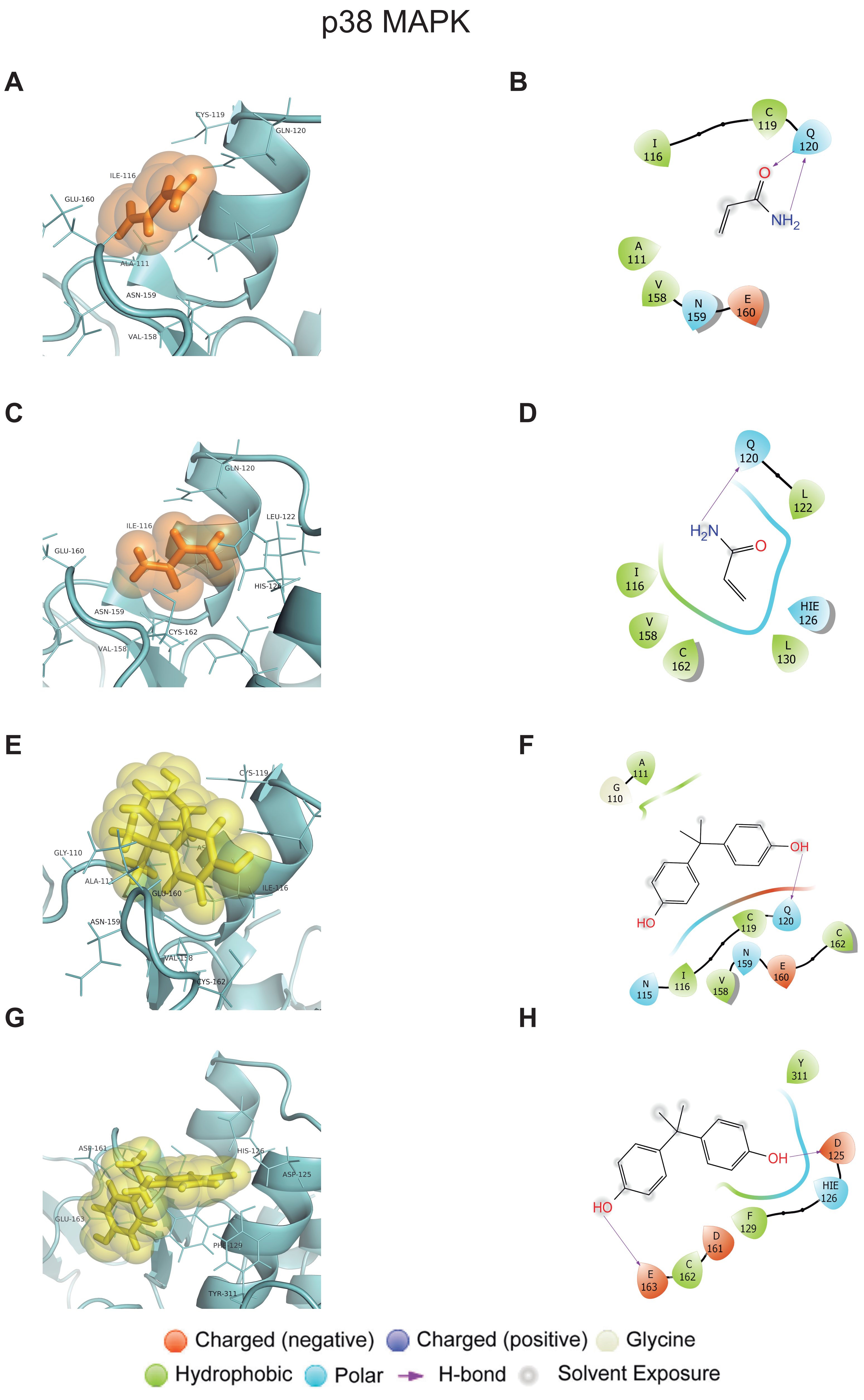

p38 MAPK
A
CYS-119
GLN-120
ILE-116
GLU-160
ALA-111
ASN-159
VAL-158
B
I 116
C 119
Q 120
O
NH2
A 111
V 158
N 159
E 160
C
GLN-120
LEU-122
ILE-116
GLU-160
HIS-126
ASN-159
CYS-162
VAL-158
D
Q 120
L 122
H2N
O
I 116
HIE 126
V 158
C 162
L 130
E
CYS-119
GLY-110
ALA-111
GLU-160
ILE-116
ASN-159
VAL-158
CYS-162
F
A 111
G 110
OH
HO
Q 120
C 119
C 162
N 159
E 160
N 115
I 116
V 158
G
ASP-161
HIS-126
ASP-125
GLU-163
PHE-129
TYR-311
H
Y 311
OH
D 125
HIE 126
HO
F 129
D 161
C 162
E 163
Charged (negative)
Charged (positive)
Glycine
Hydrophobic
Polar
H-bond
Solvent Exposure
