# Supplementary material for: Acrylamide and bisphenol A: two plastic additives increase platelet activation, via oxidative stress
Source: Front Pharmacol. 2025 Apr 30;16:1526374. doi: 10.3389/fphar.2025.1526374 (PMC12075958; doi:10.3389/fphar.2025.1526374)
Supplement: Supplementary file 1 [file DataSheet1.zip › Supplementary Figures/Figure S8.PDF]

SOD1

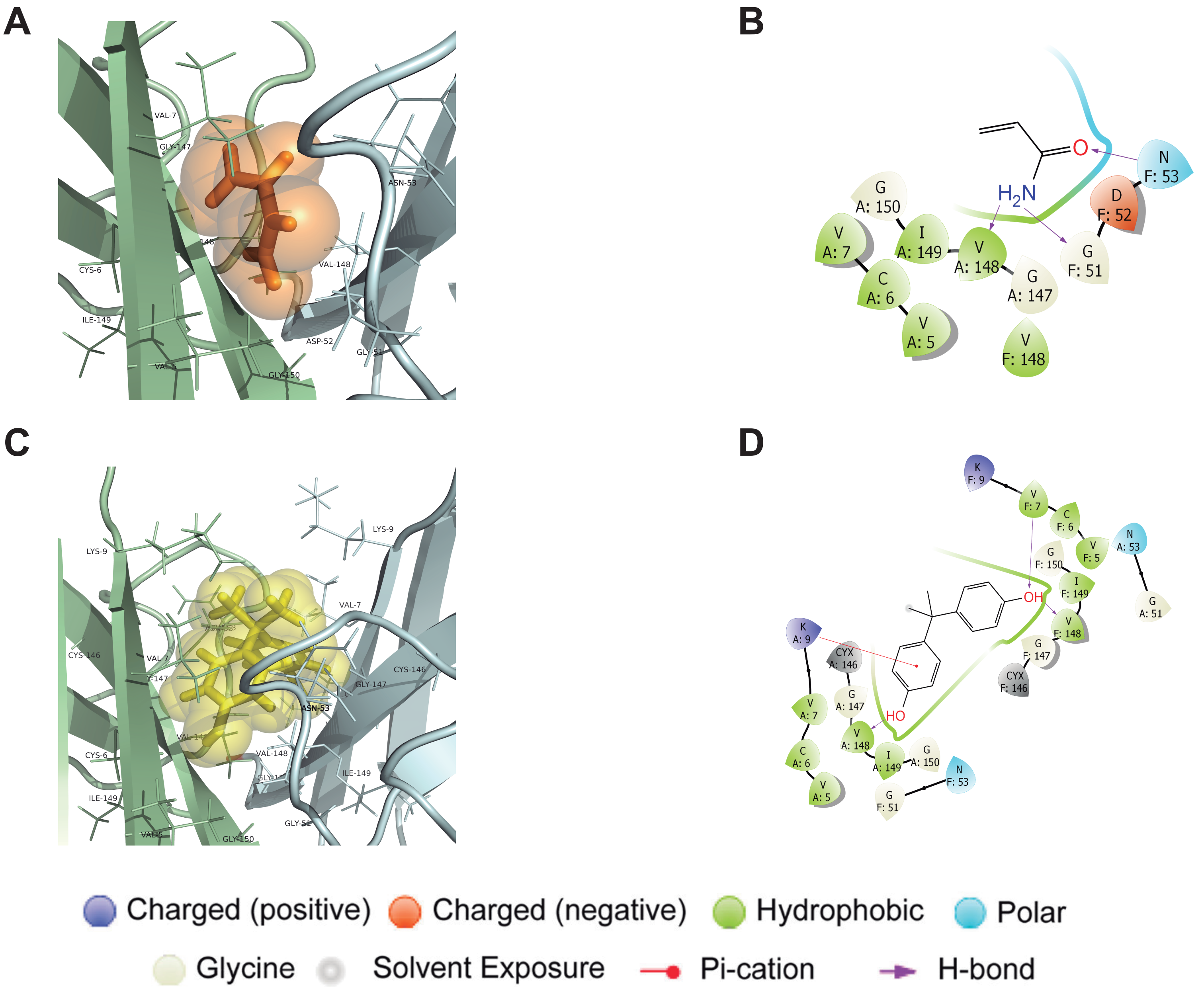
A
B
C
D
VAL-7
GLY-147
ASN-53
CYS-6
VAL-148
ILE-149
ASP-52
GLY-51
VAL-5
GLY-150
LYS-9
CYS-146
H_2N
O
OH
HO
Charged (positive)
Charged (negative)
Hydrophobic
Polar
Glycine
Solvent Exposure
Pi-cation
H-bond
