# Supplementary figures and images for: Acrylamide and bisphenol A: two plastic additives increase platelet activation, via oxidative stress
Source: Front Pharmacol. 2025 Apr 30;16:1526374. doi: 10.3389/fphar.2025.1526374 (PMC12075958; doi:10.3389/fphar.2025.1526374)

# Acrylamide

**A**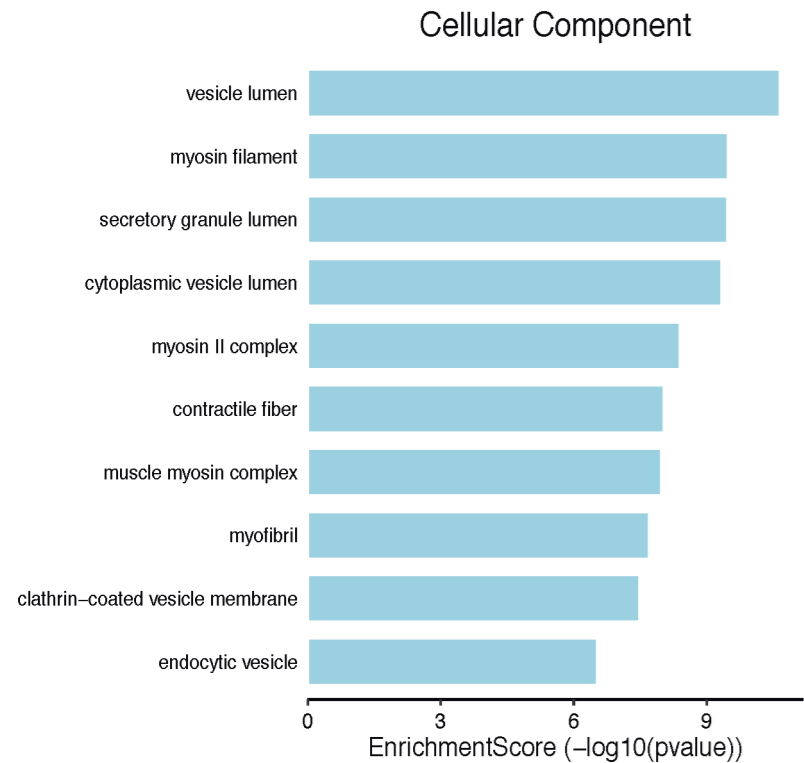**B**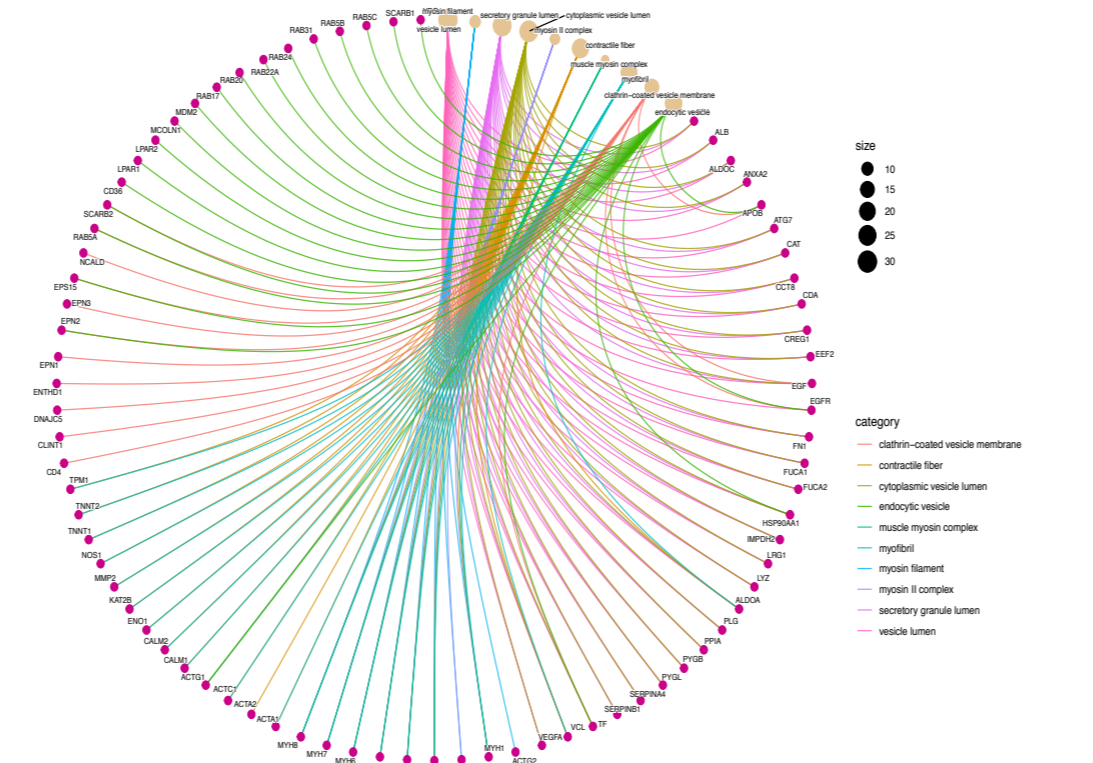**C**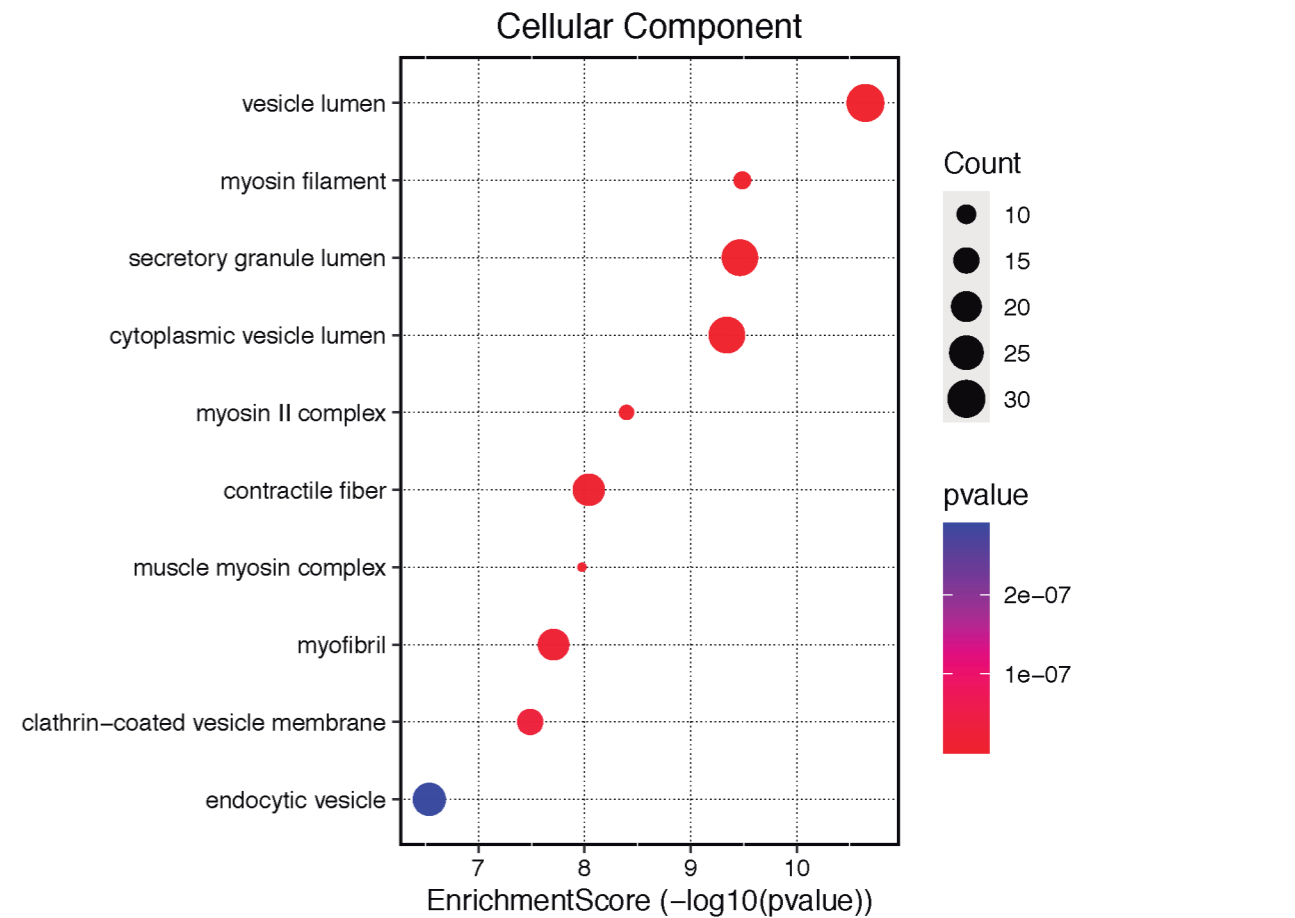**D**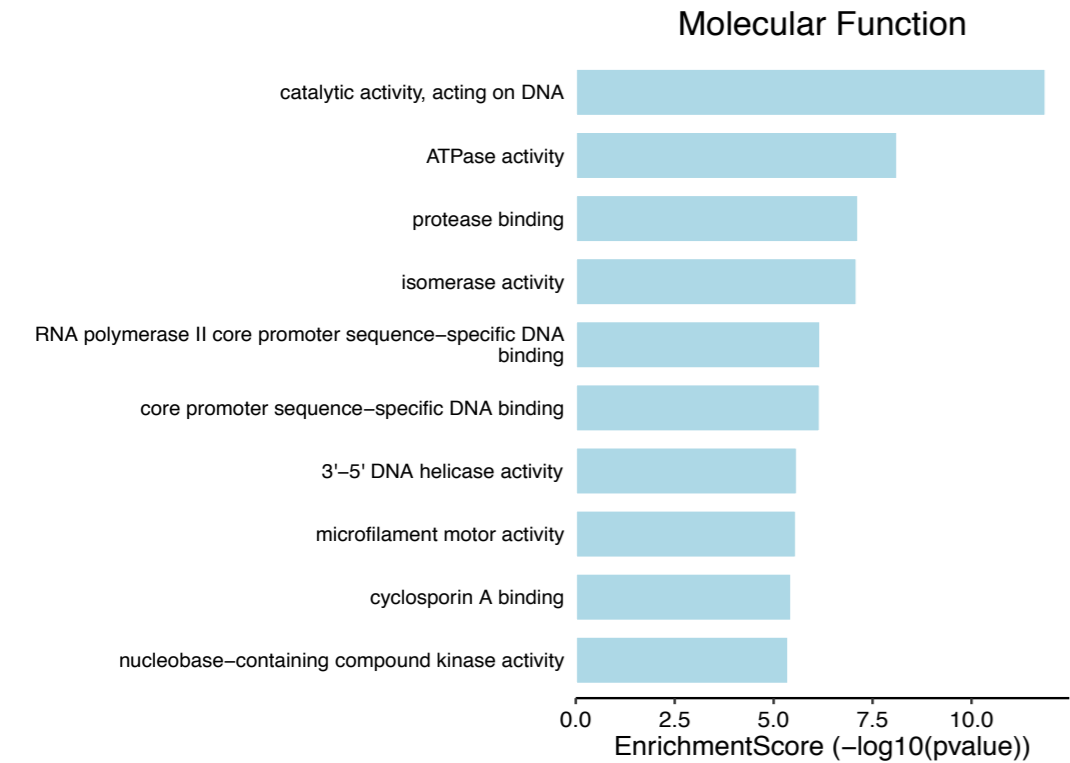**E**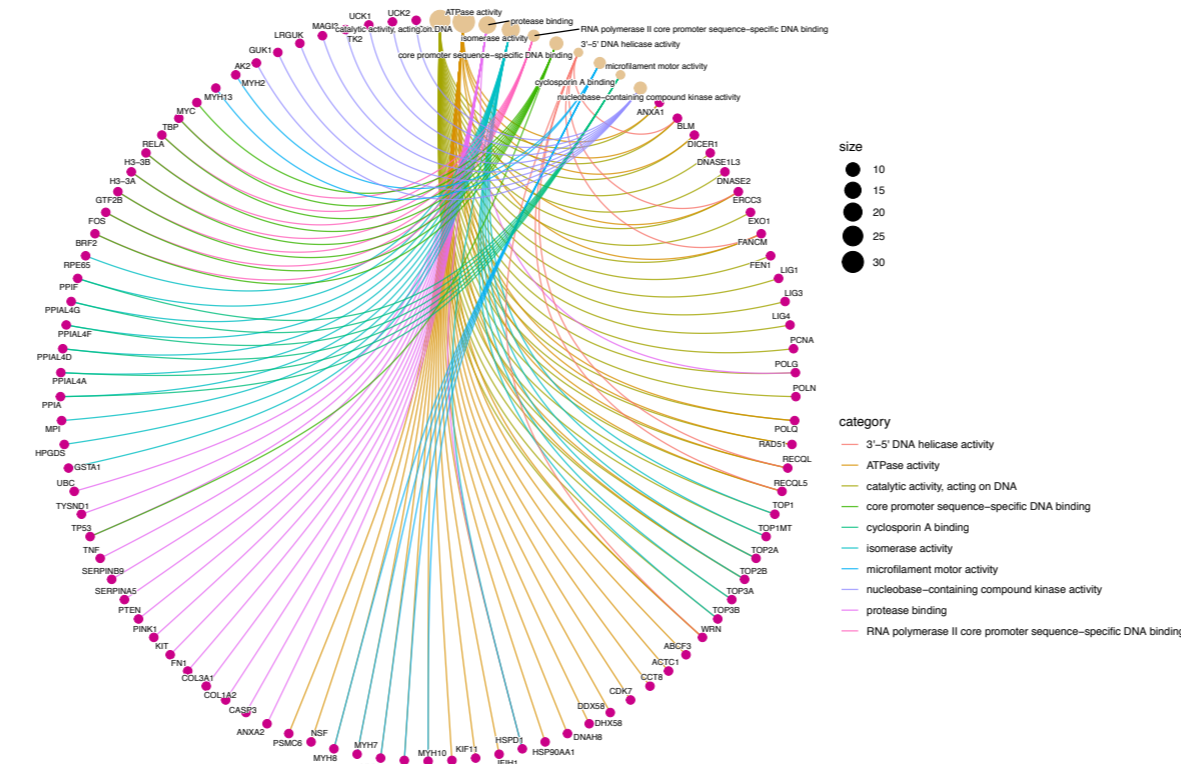**F**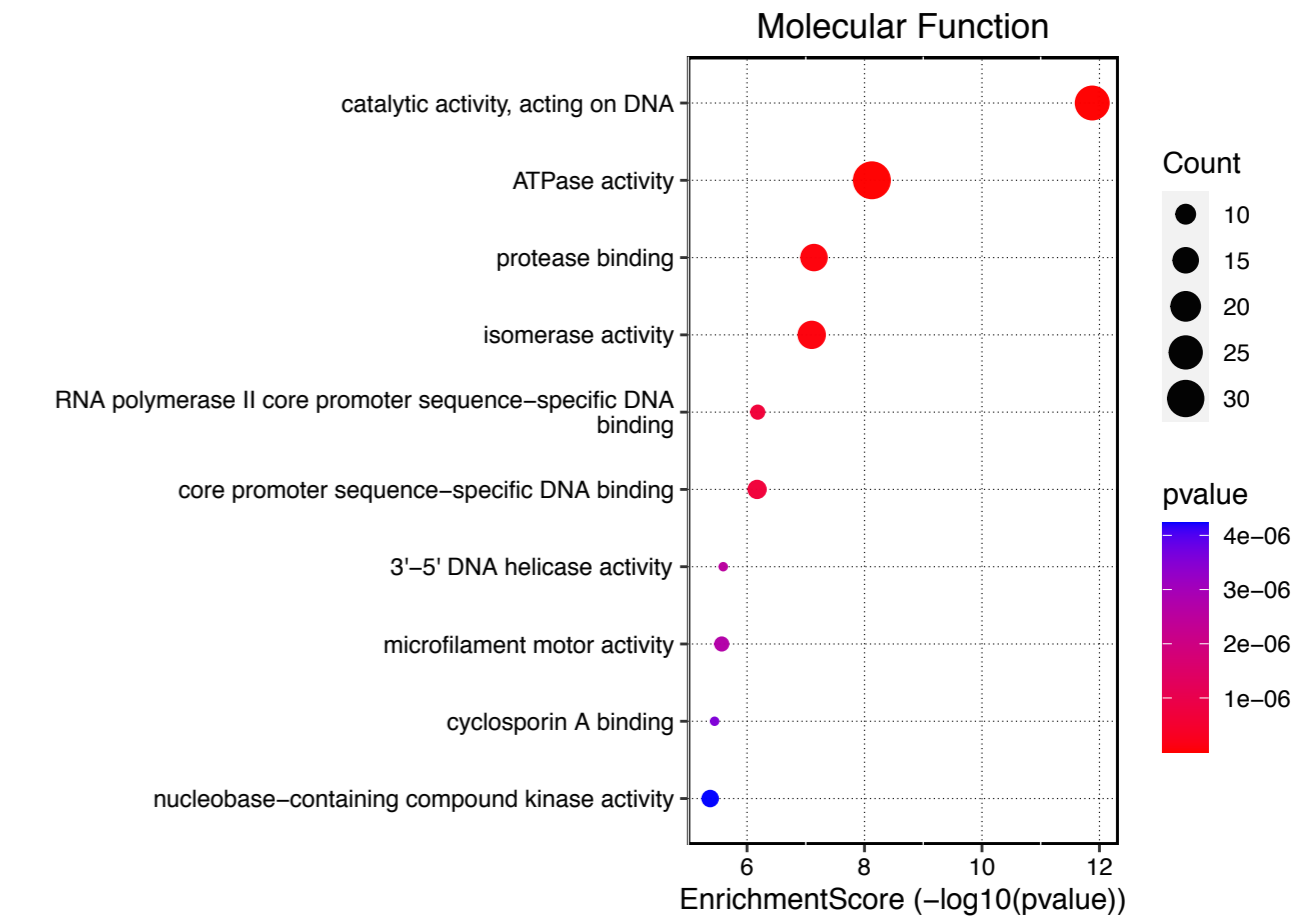

Supplement: Supplementary file 1 [file DataSheet1.zip › Supplementary Figures/Figure S1.PDF]

Acrylamide-Platelets

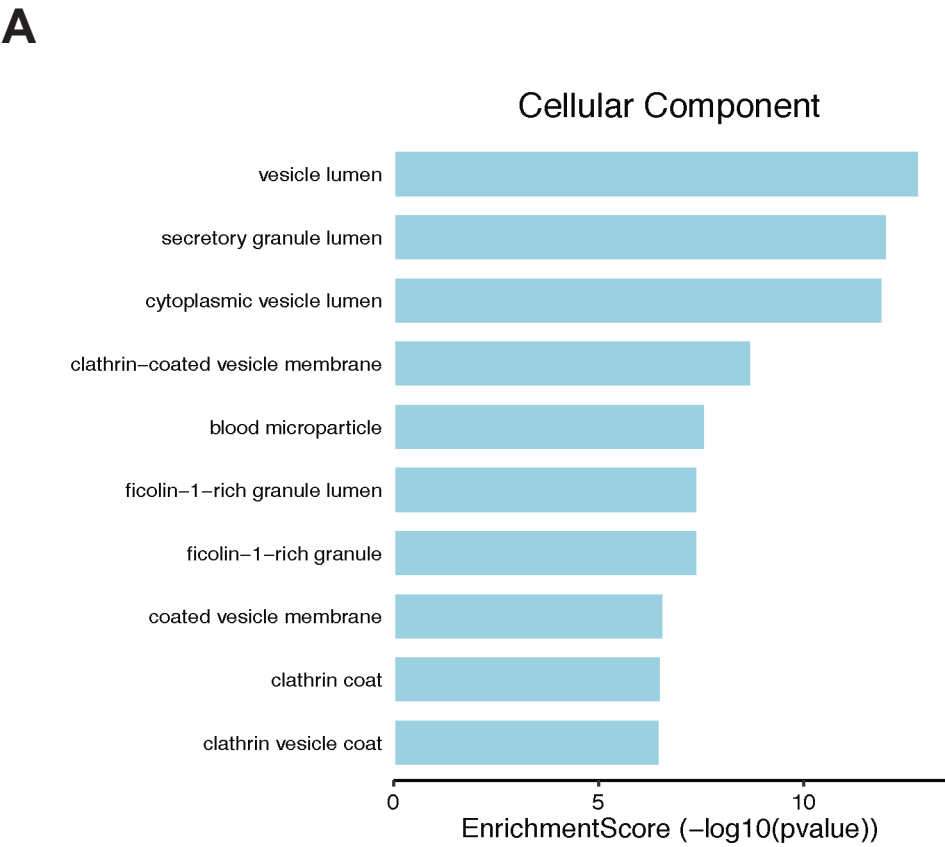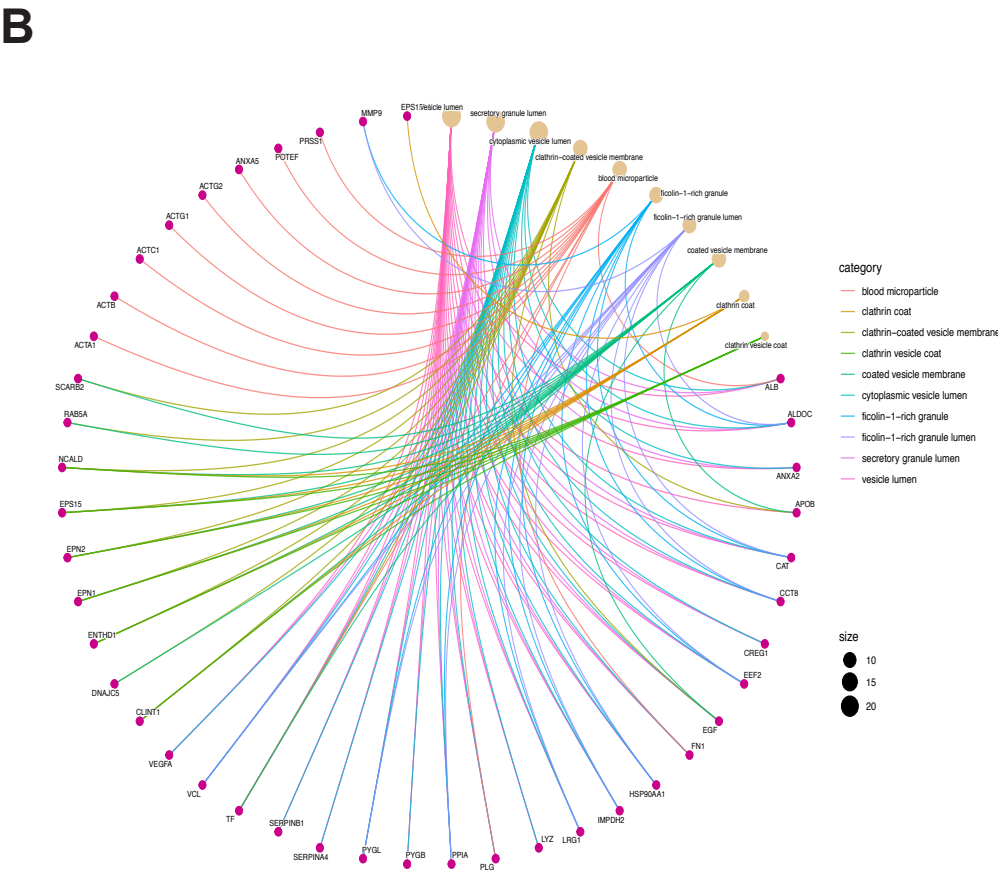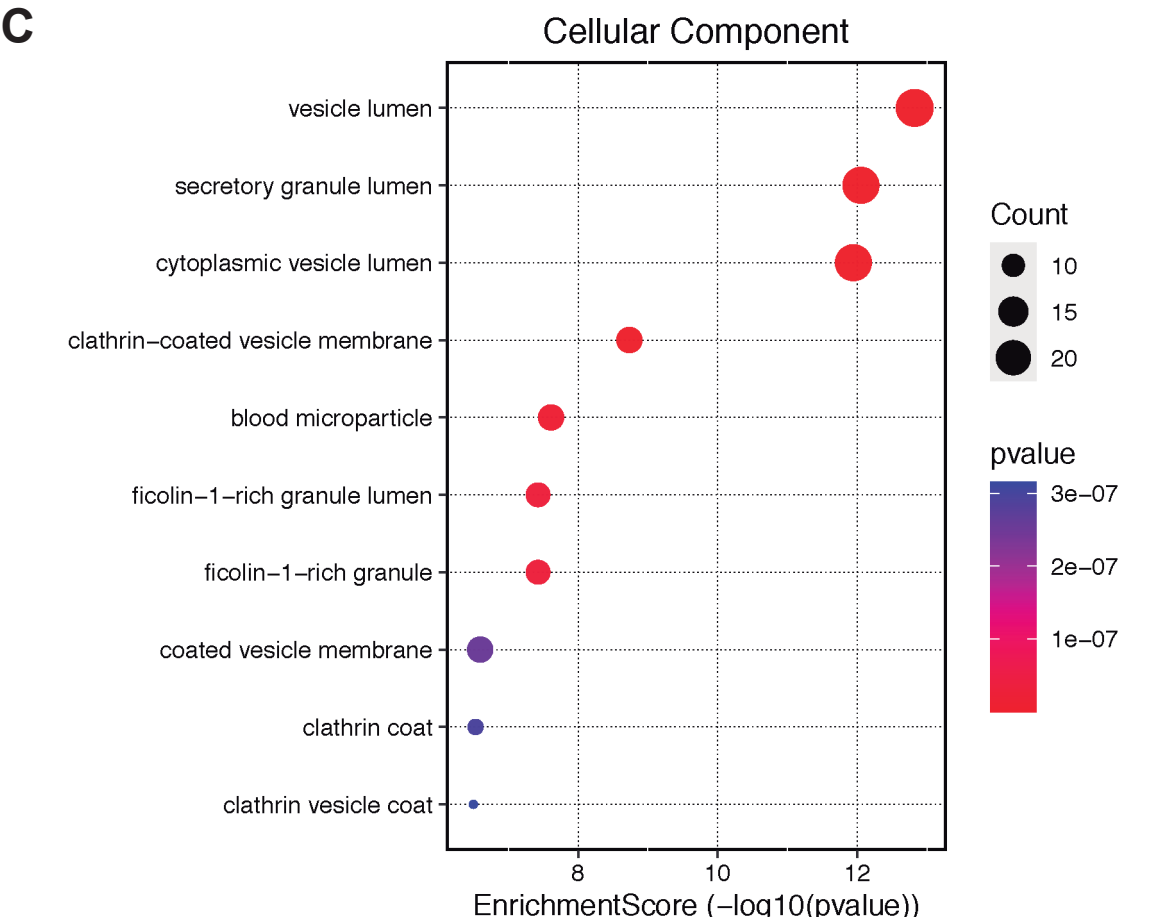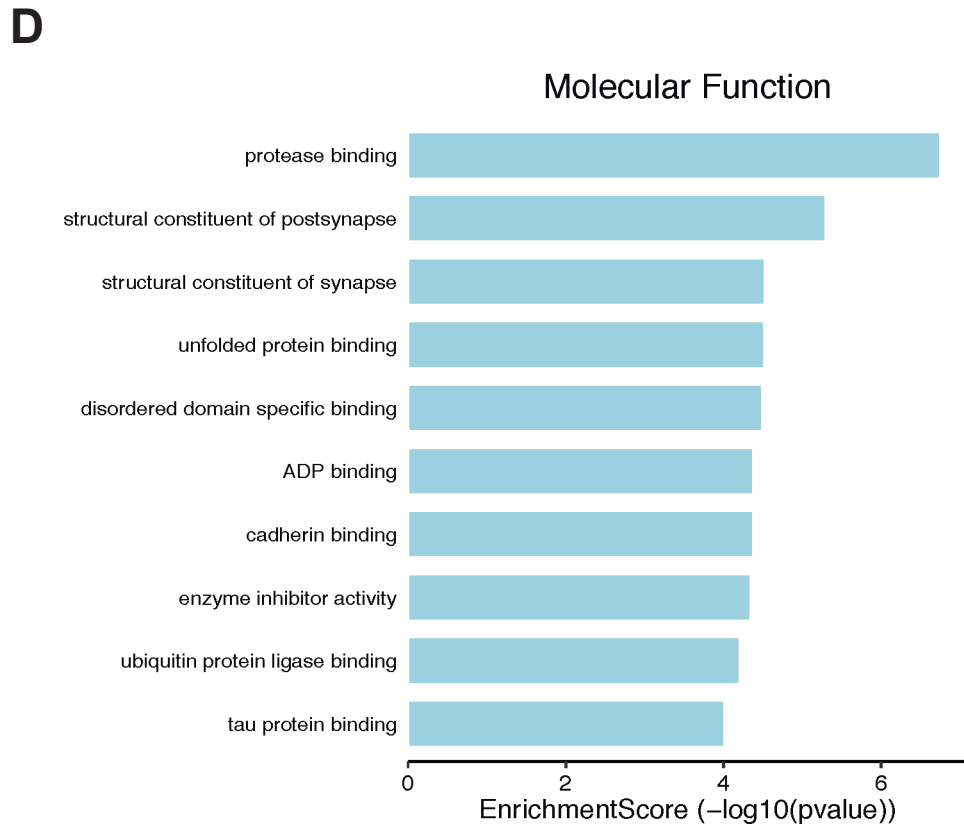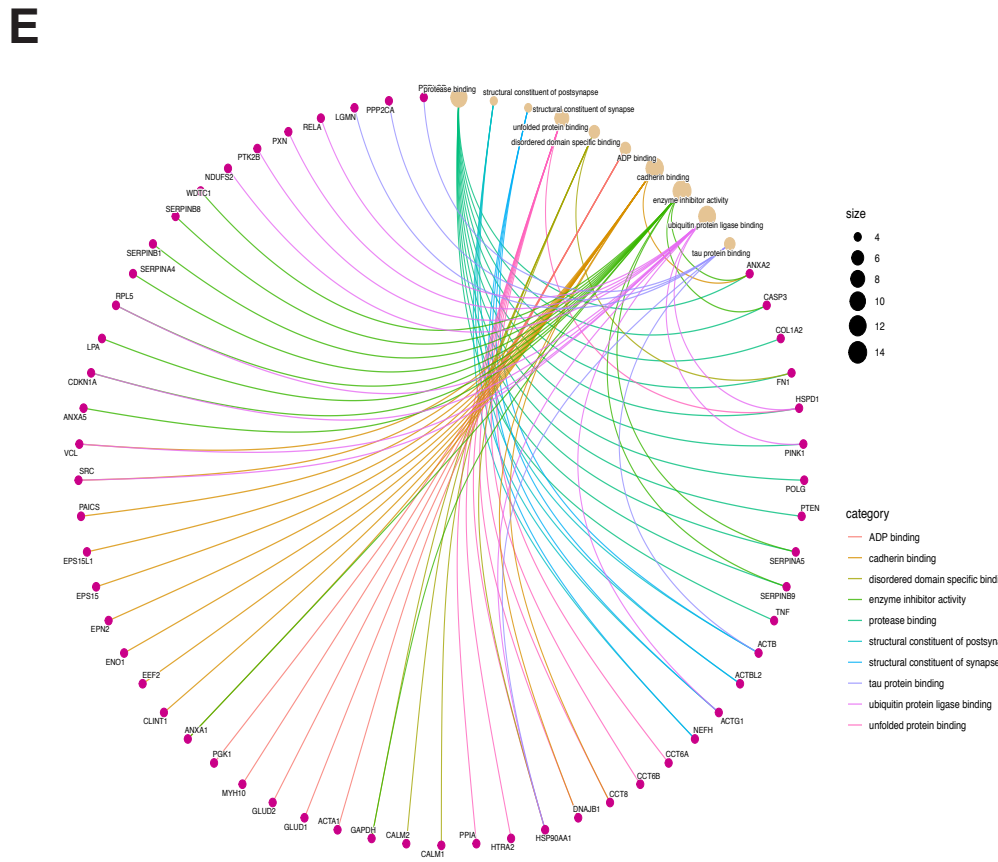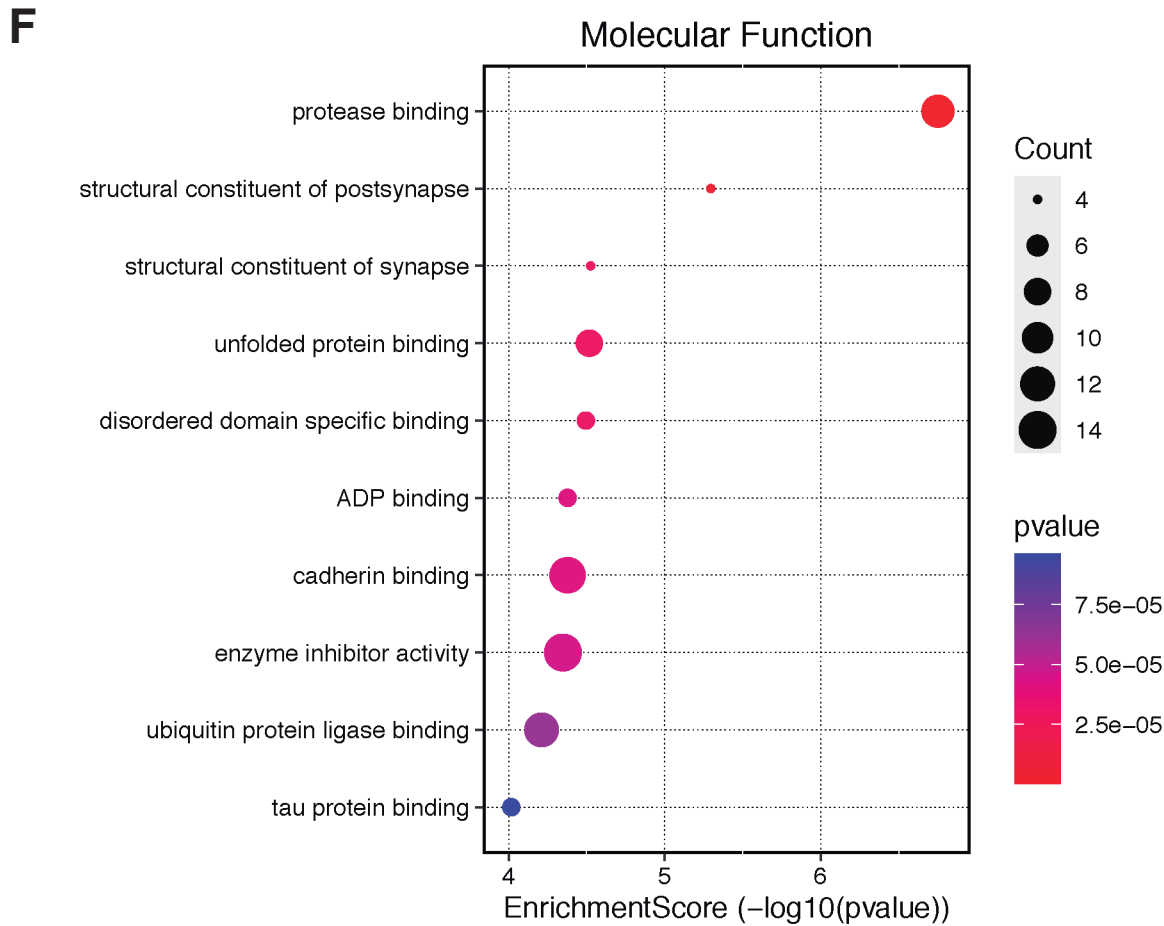

Supplement: Supplementary file 1 [file DataSheet1.zip › Supplementary Figures/Figure S2.PDF]

A

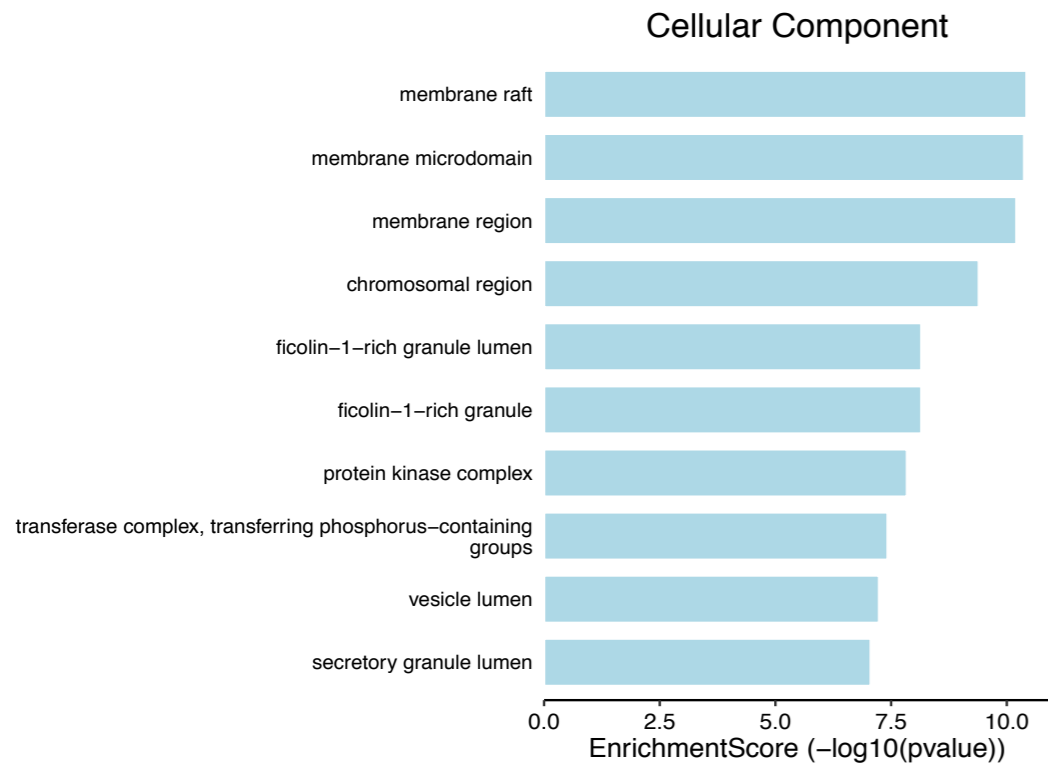

B

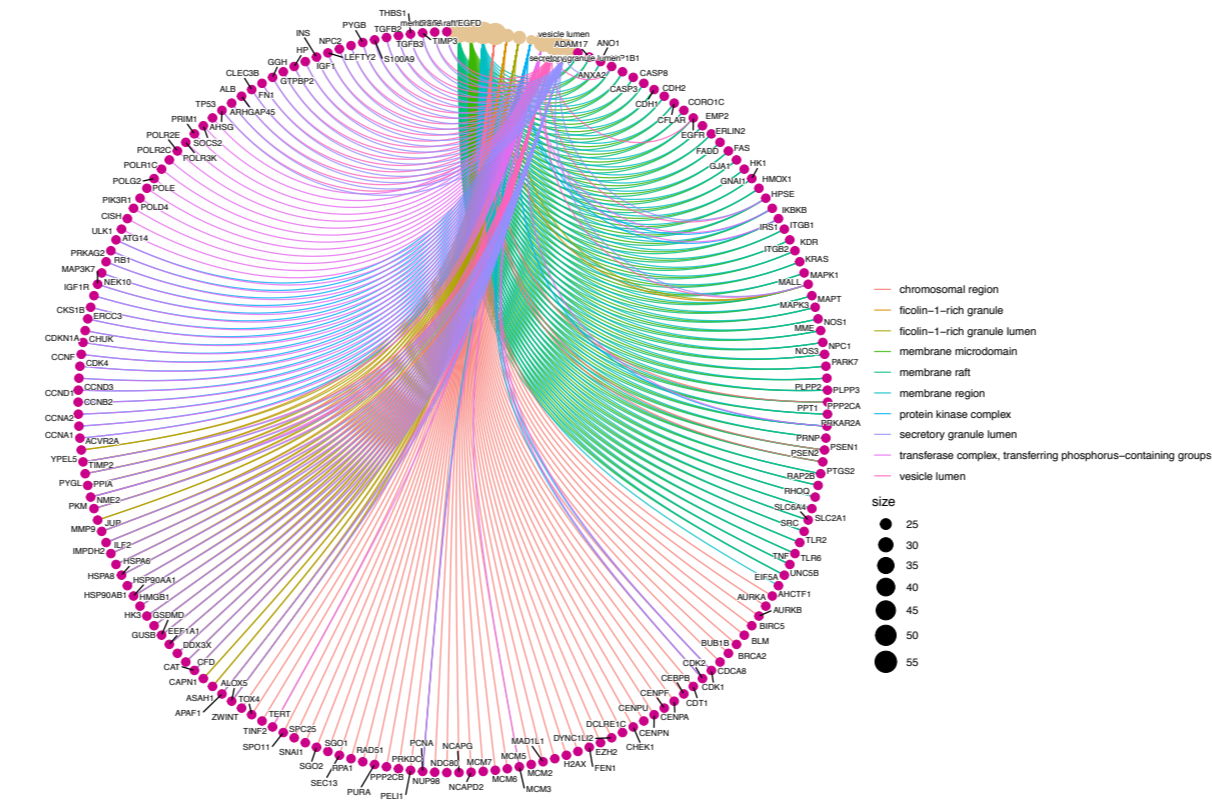

C

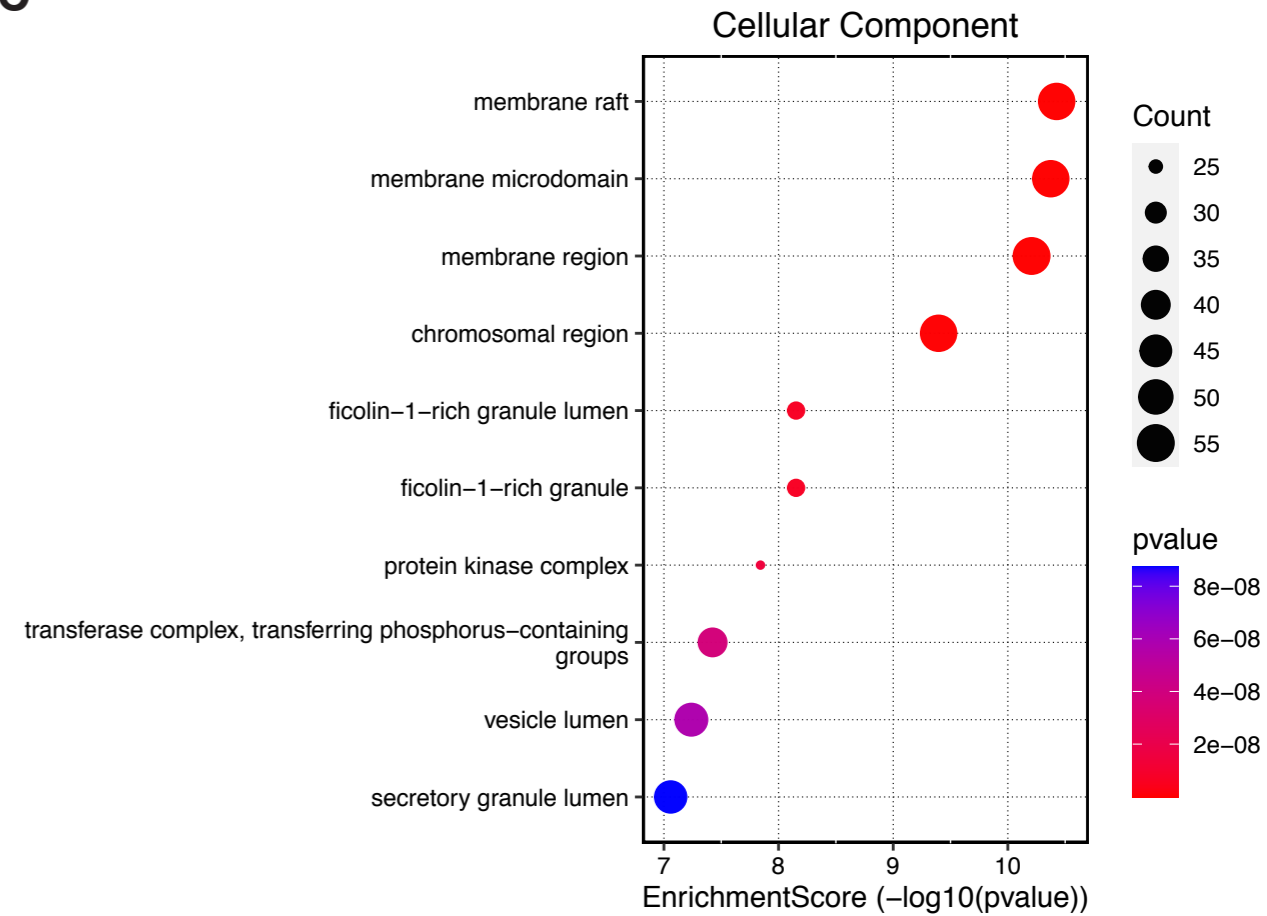

D

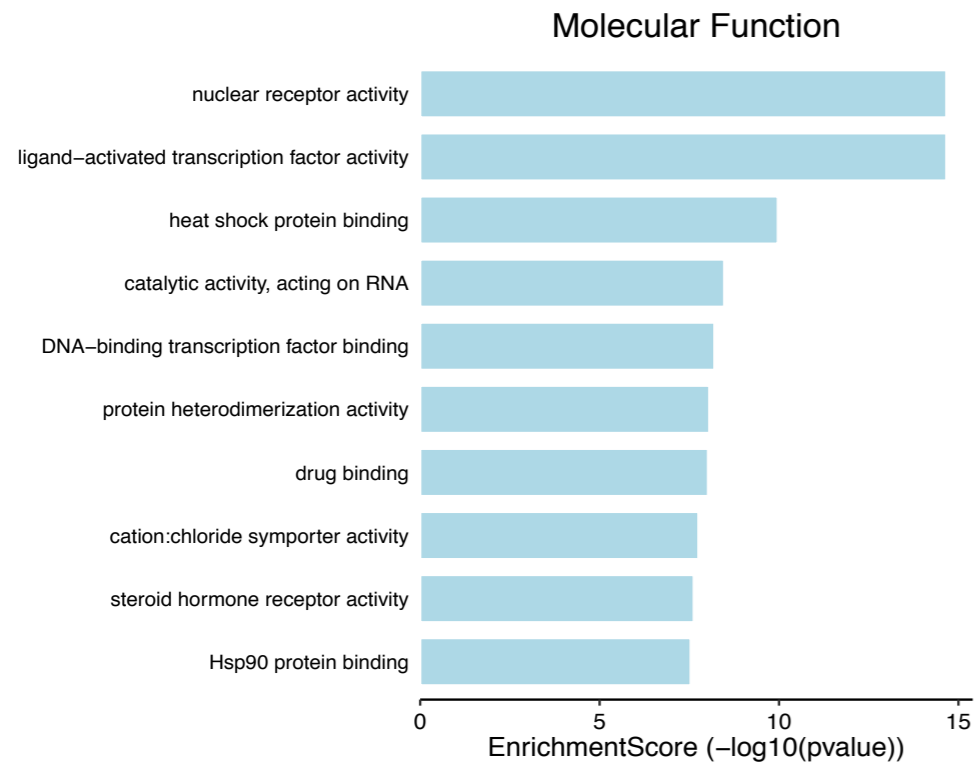

E

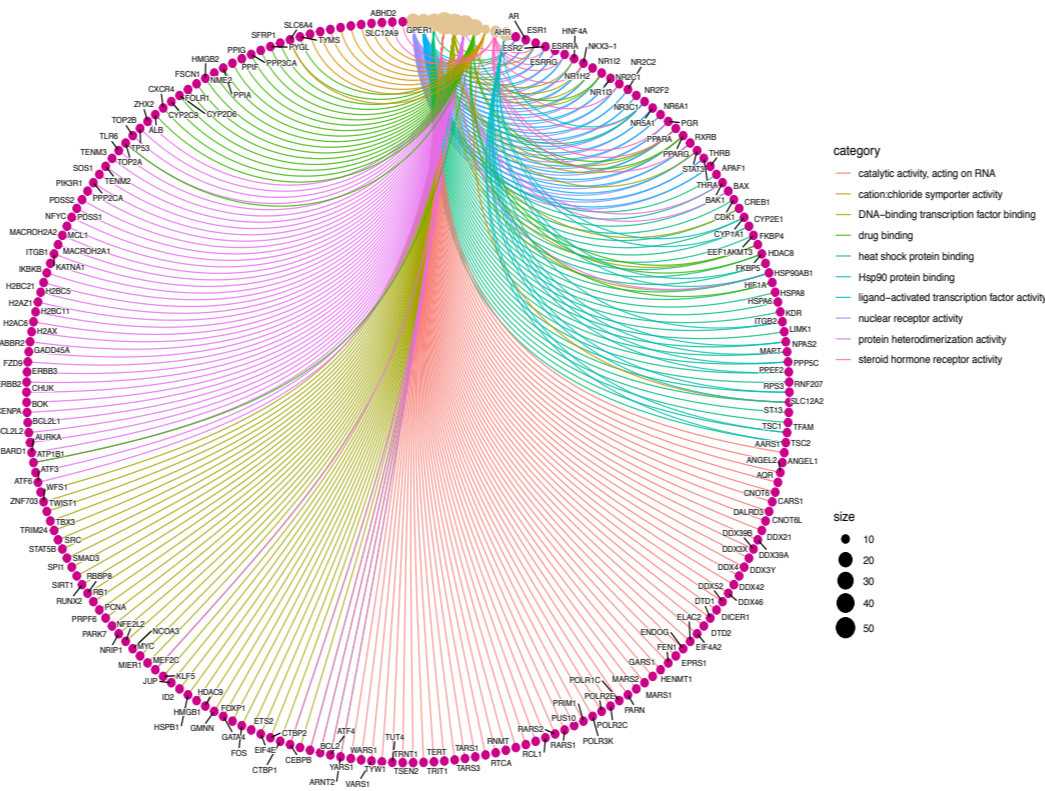

F

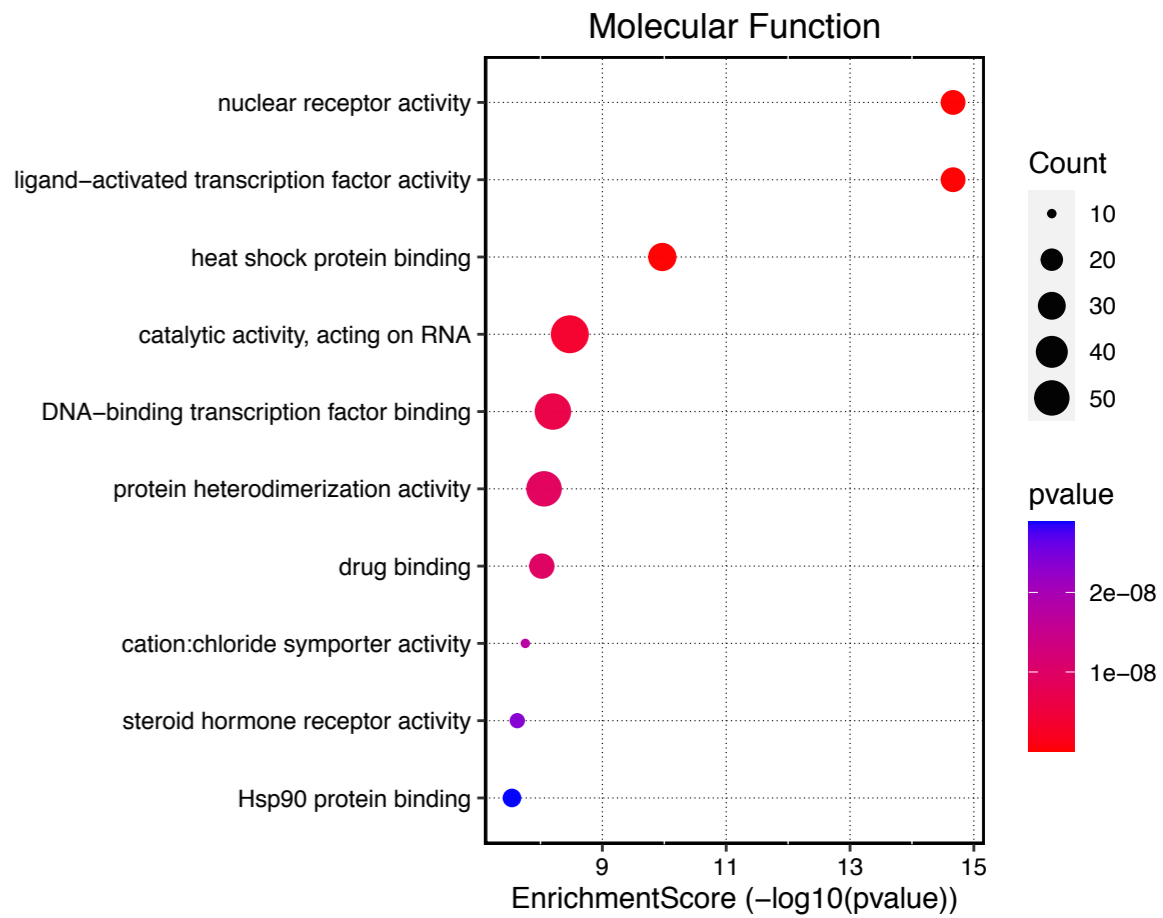

Supplement: Supplementary file 1 [file DataSheet1.zip › Supplementary Figures/Figure S3.PDF]

Bisphenol A-Platelets

A

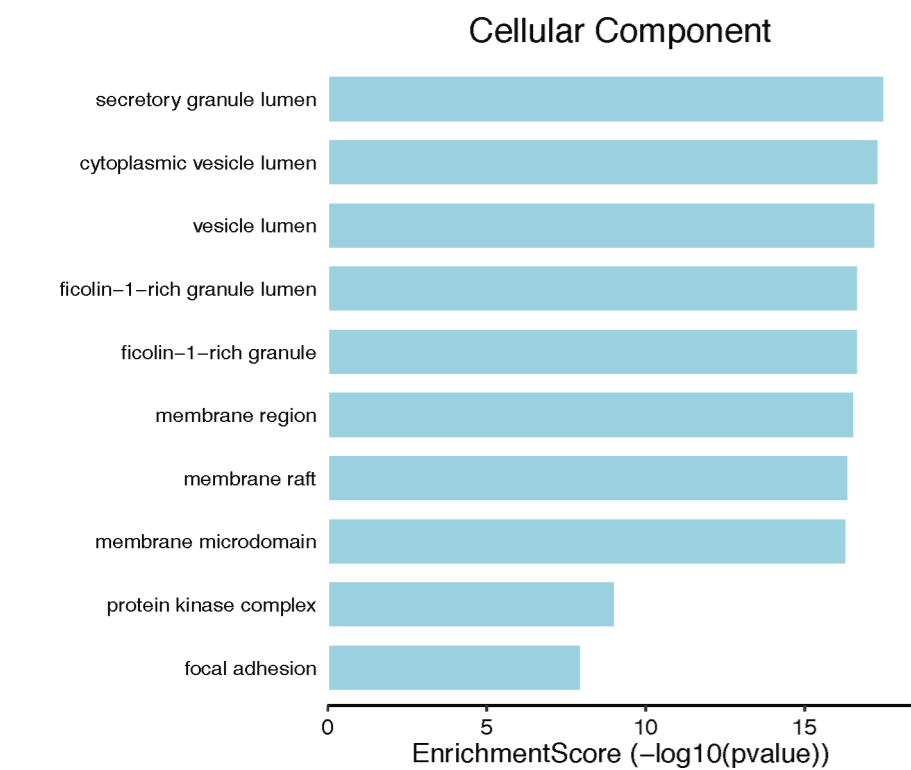

B

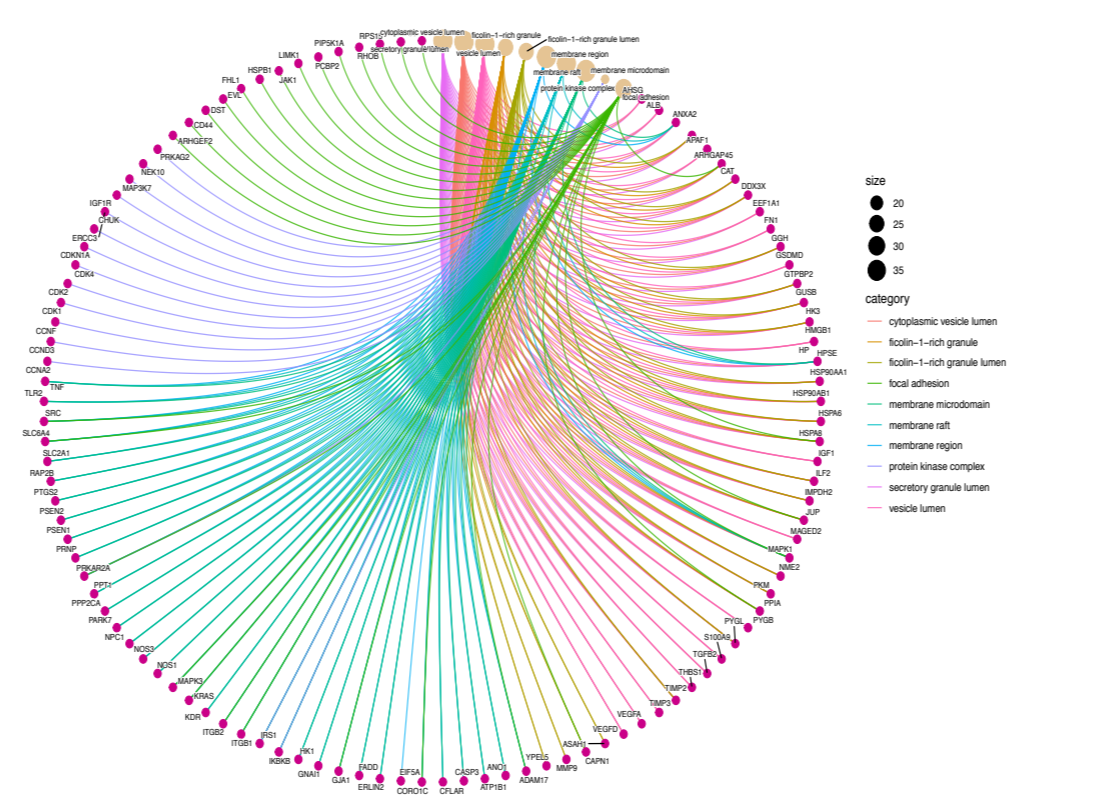

C

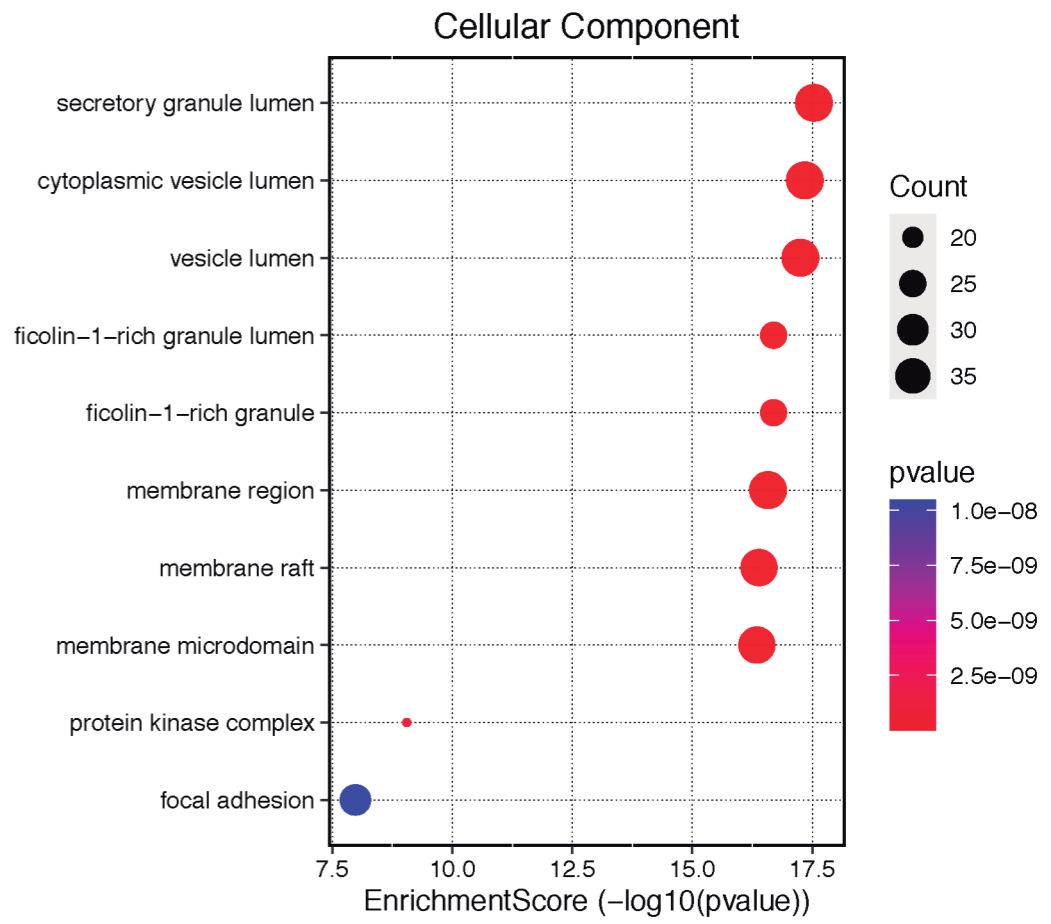

D

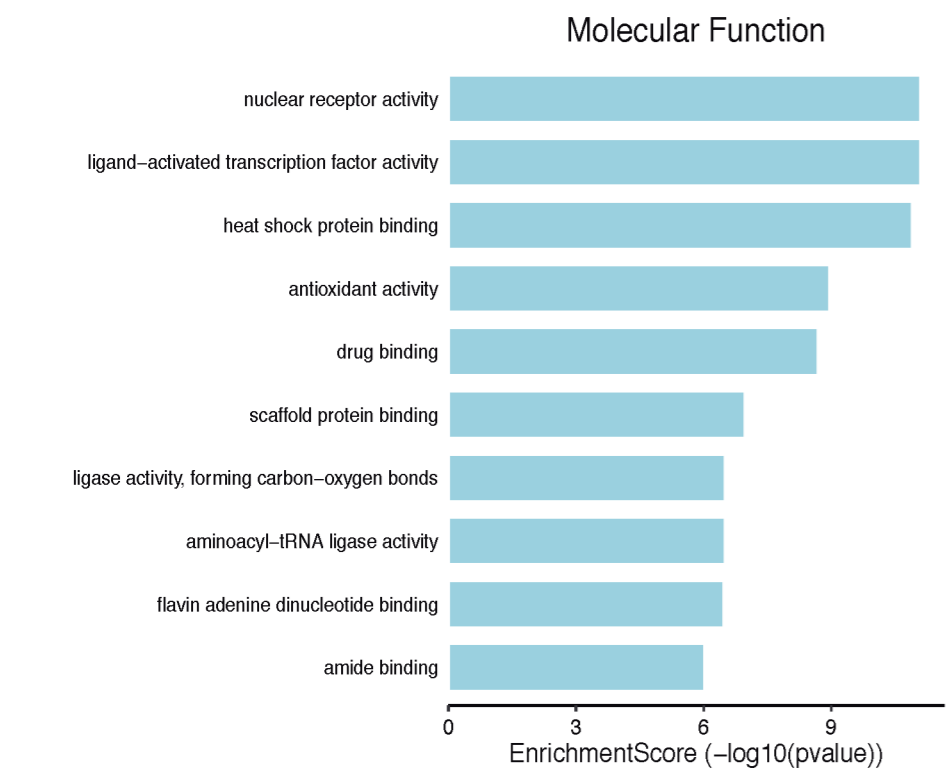

E

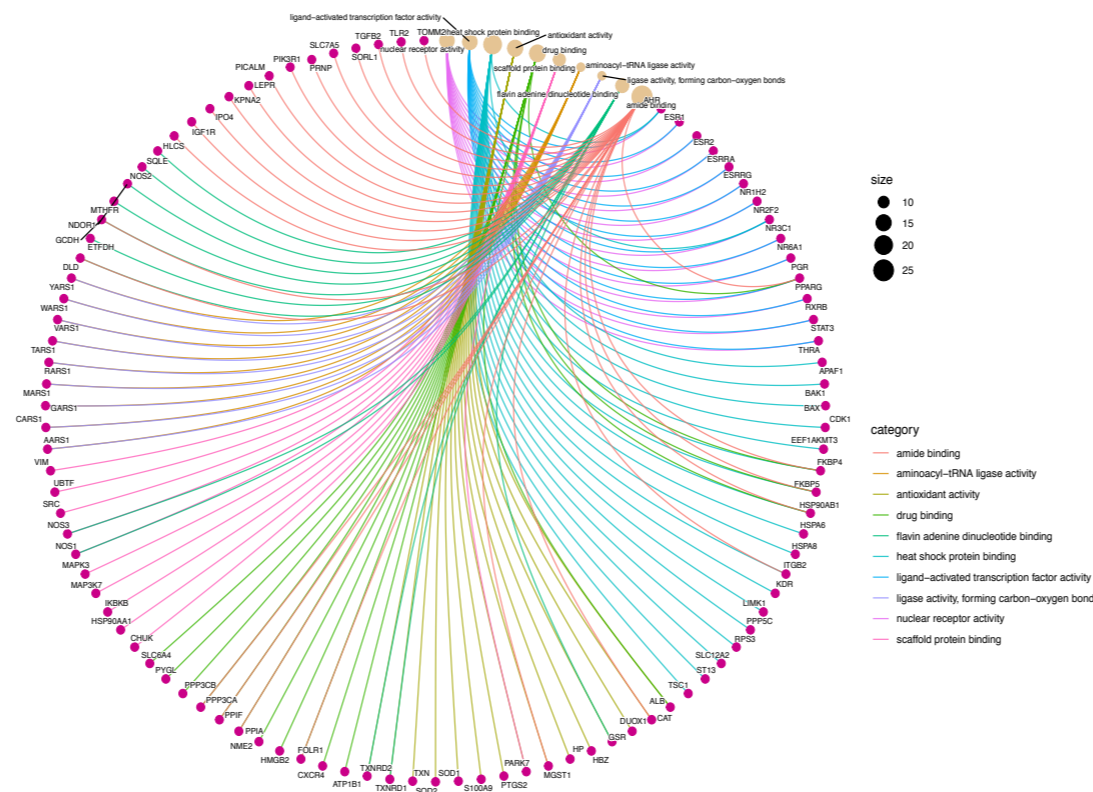

F

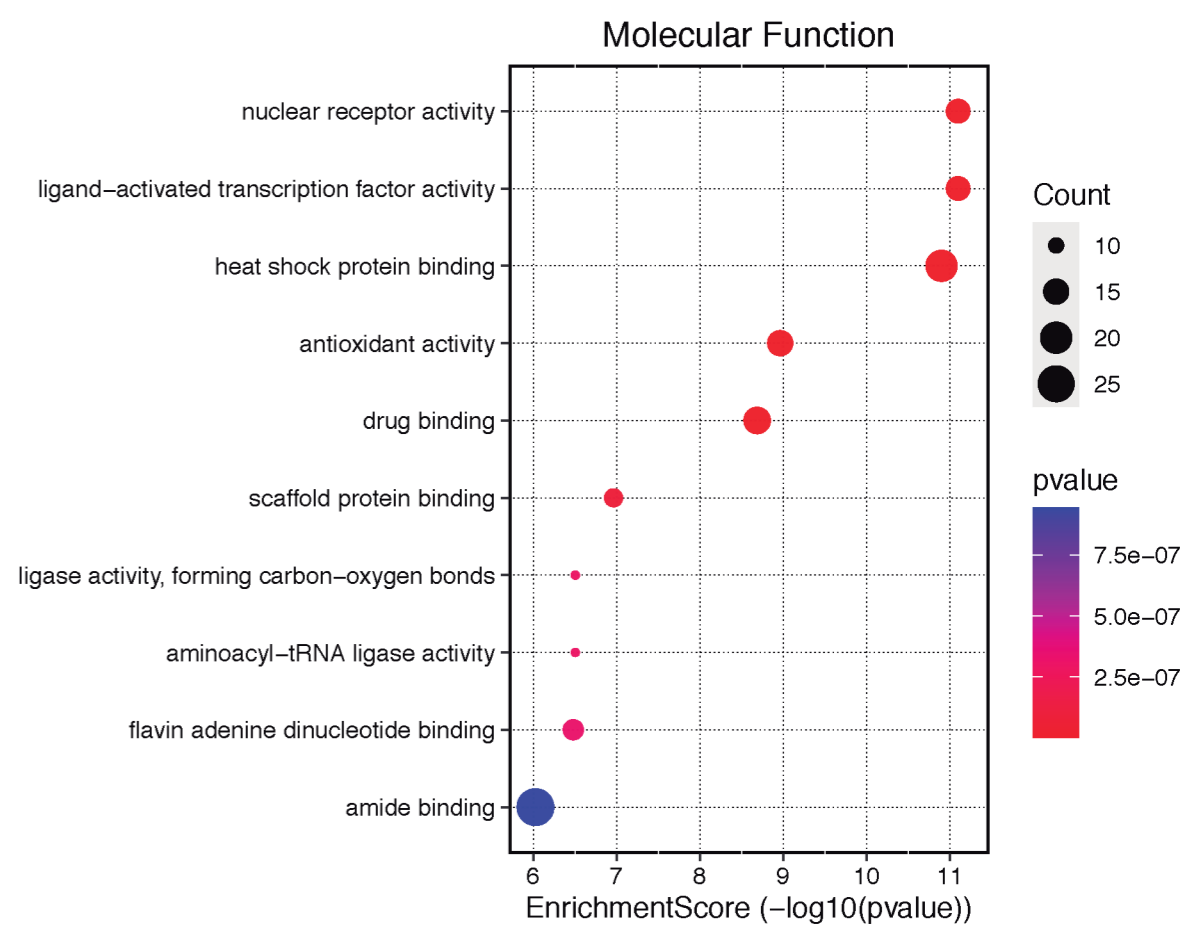

Supplement: Supplementary file 1 [file DataSheet1.zip › Supplementary Figures/Figure S4.PDF]

A

# Acrylamide

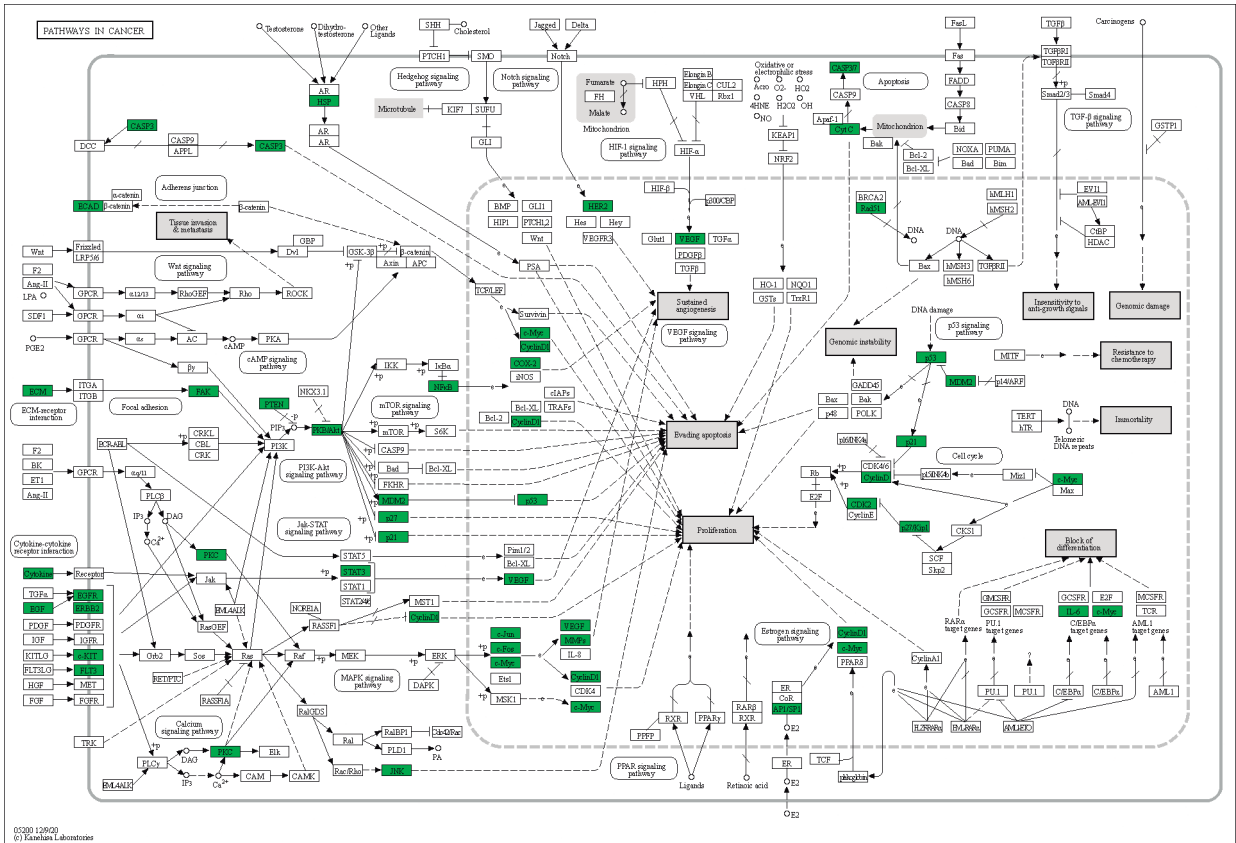

B

# Bisphenol A

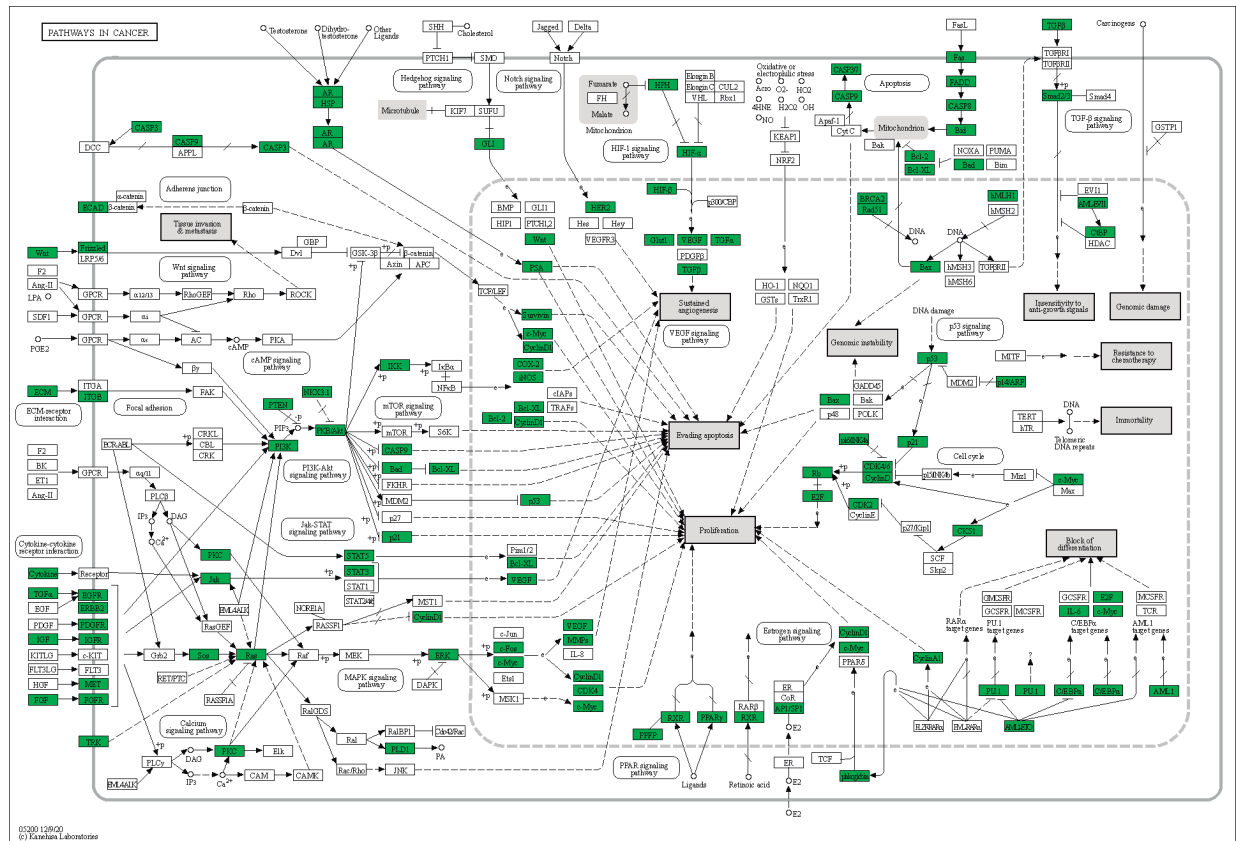

Supplement: Supplementary file 1 [file DataSheet1.zip › Supplementary Figures/Figure S5.PDF]

# PKC C1A domain

**A**

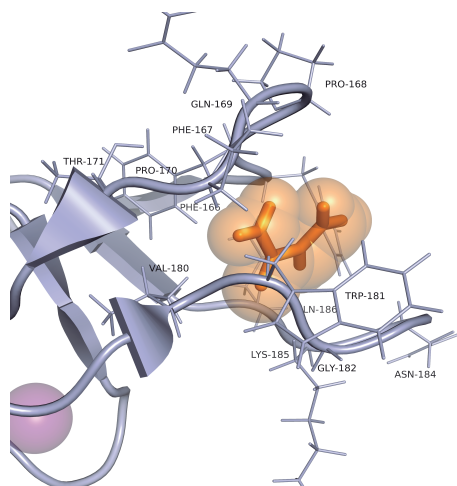

**B**

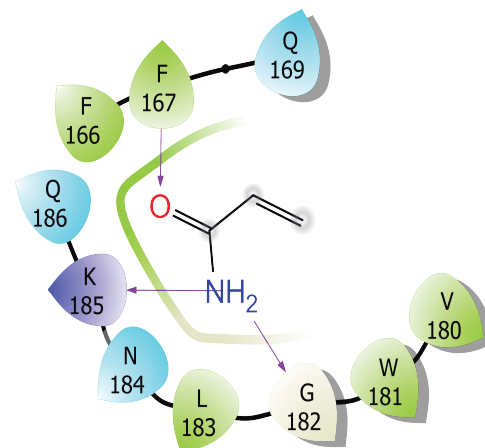

**C**

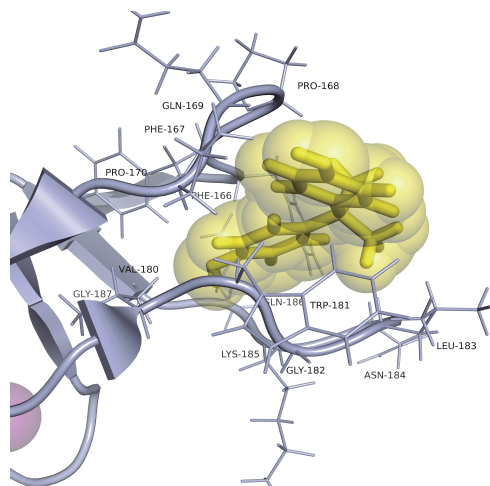

**D**

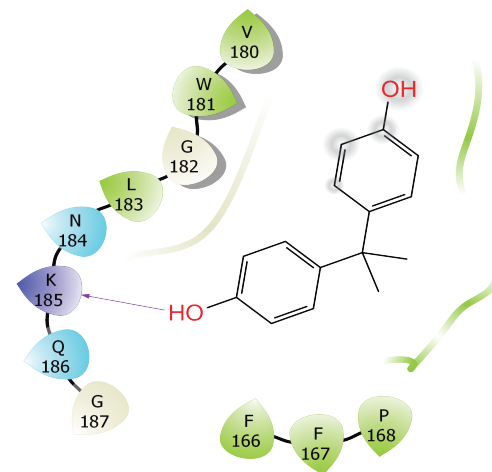

● Charged (positive) 
 ● Hydrophobic 
 ● Polar 
 ● Glycine 
 → H-bond 
 ● Solvent Exposure

Supplement: Supplementary file 1 [file DataSheet1.zip › Supplementary Figures/Figure S6.PDF]

**A**

Control

Agonist

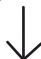

10%  
1 min

ADP 4  $\mu$ M**B**

Acrylamide

10  $\mu$ M  
25  $\mu$ M  
50  $\mu$ M

10%  
1 min

**C**

Bisphenol A

10  $\mu$ M  
25  $\mu$ M  
50  $\mu$ M

10%  
1 min

Supplement: Supplementary file 1 [file DataSheet1.zip › Supplementary Figures/Figure S9.PDF]
